# Mechanisms of therapeutic antibodies immunogenicity: infliximab oligomeric aggregates increase T-cell response and uptake by dendritic cells involving mannose-sensitive receptor CD206

**DOI:** 10.3389/fimmu.2026.1904926

**Published:** 2026-09-03

**Authors:** Maria Lteif, Myriam Nabhan, Cécile Tardif, Claire Smadja, Jennifer Mata-Orozco, Stéphane Hua, Bernard Maillère, Marc Pallardy, Isabelle Turbica

**Affiliations:** 1 Université Paris Saclay, Institut National de la Santé et de la Recherche Médicale (INSERM), Inflammation, Microbiome, Immunosurveillance, Faculté de Pharmacie, Orsay, France; 2 Université Paris Saclay, Centre National de la Recherche Scientifique (CNRS) UMR 8612, Institut Galien Paris Saclay, Orsay, France; 3 Université Paris-Saclay, Commissariat à l’énergie Atomique (CEA), Institut National de Recherche pour l’Agriculture, l’alimentation et l’Environnement (INRAE), Département Médicaments et Technologies pour la Santé, SIMoS, Gif-sur-Yvette, France

**Keywords:** aggregation, dendritic cells, immunogenicity, internalization, T cell repertoire

## Abstract

**Introduction:**

Aggregation of therapeutic antibodies (Ab)s is now recognized as a major product-related factor associated with increased potential of immunogenicity. Regulatory agencies have established limits for the presence of (sub)visible particles in commercialized products. These particles are considered a potential risk factor for the development of unwanted immune responses. However, little is known concerning the role of oligomeric Abs aggregates of nanometric size in Abs’ immunogenicity. The objective of this work was to better understand the role and the mechanisms of T-cell response to nanometric Ab aggregates using infliximab (IFX) as a model.

**Method:**

We produced nanosized IFX aggregates by exposing native IFX to ultraviolet light. These aggregates were characterized using size exclusion chromatography (SEC), dynamic light scattering (DLS) and field flow fractionation coupled with a multi-angle light scattering detector (FFF-MALS). We then investigated the impact of IFX aggregation on its uptake by dendritic cells and the subsequent activation of IFX-specific T-cell responses.

**Results:**

Using a human in vitro autologous dendritic cell (DC)/T-cells coculture assay, we showed that aggregation increased the frequency of responsive T cells to IFX. Moreover, we observed exclusive T-cell responses to IFX aggregates and different TCR clonotypes responding to each form of IFX. Using fluorescent native or aggregated IFX, we found a significantly higher internalization rate of aggregated IFX by DCs in a mannose-dependent manner. Confocal microscopy experiments showed that aggregates were rapidly internalized and associated with markers of early endosomes and lysosomes as well as antigen presentation molecules, compared to the native form of IFX.

**Discussion:**

Our results suggest that internalization of IFX oligomeric aggregates via the mannose receptor pathway significantly contributes to enhanced IFX uptake by DCs and increased routing into the degradative pathway for processing into peptides. Increased presentation of immunogenic peptides may contribute to the augmentation of T-cell recruitment with aggregates compared to the native form.

## 1 Introduction

Tumor necrosis factor (TNF)-alpha (α) is a potent pro-inflammatory cytokine involved in the physiopathology of chronic inflammatory conditions and autoimmune diseases such as rheumatoid arthritis, systemic lupus erythematosus, inflammatory bowel disease, and multiple sclerosis (1). Although highly effective, some patients don’t respond to anti-TNFα biotherapeutics (primary non-response) or lose response over time (secondary non-response) (2). One of the major cause of secondary non-response is the formation of anti-drug antibodies (ADA) with neutralizing properties (3). ADAs (neutralizing or non-neutralizing) can also impact antibodies (Abs) pharmacokinetics by forming immune complexes with the drug enhancing its clearance from circulation (4). Infliximab (IFX), a chimeric monoclonal Ab IgG1, was the first anti-TNFα Ab approved for the treatment of Crohn’s disease and is currently used for the management of many other inflammatory diseases (5). However, IFX is immunogenic with 17% to 65% of treated patients developing ADA 10 to 14 days up to months after treatment, depending on the underlying disease that warranted IFX’s administration (6–8). IgG1 and IgG4 are the main ADAs detected in patients’ sera (9) suggesting a central role for CD4+ and isotype switching.

ADA response may result from the generation of an adaptive immune response with four critical steps. First, Abs or immune-complexes are internalized by antigen-presenting cells APCs such as dendritic cells (DC)s through macropinocytosis, phagocytosis or (non-)receptor-mediated endocytosis. Then, internalized proteins are sorted in early endosomes to be either recycled to the plasma membrane or to follow their journey to lysosomes for further degradation into peptides (10, 11). APCs present these peptides at their surface in association with major histocompatibility complex (MHC) class II molecules to activate specific naive CD4+ T cells resulting in activation of B cells and the production of high-affinity ADA (12). Abs properties, particularly their glycosylation, a critical post-translational modification influencing their functional activity, might impact their internalization pathways and, consequently, their immunogenicity (13–15).

Protein aggregation is a process in which proteins misfold and assemble to form a variety of soluble oligomers and insoluble aggregates (16). Aggregates are typically classified based on several criteria, including size, reversibility, conformation, and morphology (17). The presence of protein aggregates in the administrated preparations is now well accepted and regulatory agencies consider visible and sub-visible aggregates as potential risk factors for inducing unwanted immunogenicity of therapeutic proteins (18, 19). Since aggregates ≤10 μm are known to have a greater risk of immunogenicity, the U.S. Food and Drug Administration (FDA) has tightened the regulatory scrutiny of aggregates below 10 μm in biologic product (20). However, once commercialized, pH or temperature variation or mechanical stresses occurring during handling, shipping, and storage of therapeutic proteins can impact their stability, thus favoring aggregation (21, 22). Oxidation caused by light exposure is also an important degradation pathway of therapeutic Abs resulting in Ab’s aggregation (23). Recent studies have evidenced the presence of nanometer, submicron, and micron protein particles in intravenous saline bags that could inadvertently be delivered to patients (24). Pardeshi et al. found that subvisible aggregates of IFX could activate *in vitro* innate and adaptive immune responses (25).

Several hypotheses have been developed to understand the mechanisms of aggregates immunogenicity. *In vivo* and *in vitro* studies have shown that aggregates could act as danger signals resulting in DC activation facilitating a T-cell-dependent response (26, 27). On the other hand, some Ab aggregates such as freeze/thaw-induced aggregates did not activate monocyte-derived DCs (moDCs), nor had any effect on the CD4 T-cell response (28). This was also the case of rituximab preparations containing less than 3% of subvisible aggregates, whereas trastuzumab preparations containing the same proportion (<3%) of aggregates resulted in increased CD86 and HLA-DR expression on moDCs’ surface and favored T-cell proliferation and IL-2 and IL-10 secretion (29). Nabhan et al. confirmed earlier findings regarding the presence of IFX-specific T cells in blood of healthy donors and showed that heat-stress generated aggregates of IFX leads to an enhanced T-cell response, characterized by a higher frequency of pre-existing specific T cells, compared to native IFX (30).

The presence in the T cell repertoire of CD4+ T cells recognizing epitopes within biotherapeutics is a prerequisite for the development of the immune response. The latter is initiated by the internalization and processing of the biotherapeutic by APCs (31–33). However, little is known concerning mechanisms of aggregates internalization by DCs and T-cell response involved in anti-aggregates immune responses. In this work, we investigated the presence of a T-cell repertoire in response to nanosized UV-stressed IFX aggregates (IFX UV) and studied the relevance of exposed mannosylation on IFX aggregates as a ligand for their internalization into DCs, two crucial factors for initiating a T-cell response. Human DCs were used as they are considered the most efficient APCs (34). Our results showed the existence of a naïve CD4+ T cell repertoire recognizing IFX in its native (IFX N) or aggregated form (IFX UV), with distinct clonotypes, and suggested a central role of the mannose sensitive receptors pathway for generating T-cell response to IFX aggregates via an increase uptake of aggregates by DCs.

## 2 Results

### 2.1 IFX aggregates characterization

**Figure 1:**
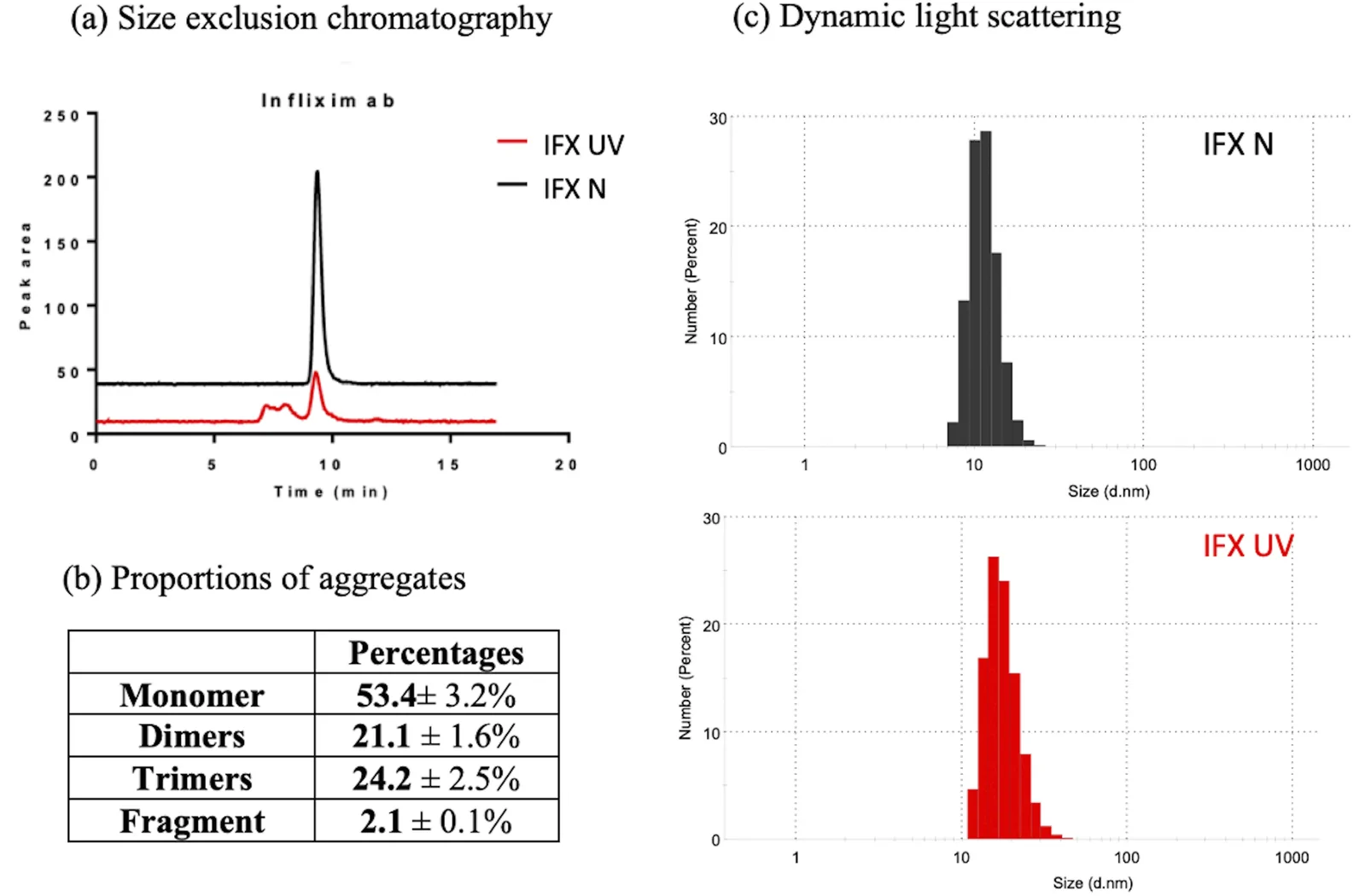
Infliximab aggregates characterization. Native (N) (1mg/ml) and aggregated (UV) infliximab (IFX) (1mg/ml) were characterized by physicochemical methods: **(a)** chromatogram overlay from size exclusion chromatography (SEC) of IFX-N (dark line) and IFX-UV (red line) (representative result of one batch); **(b)** percentages of different species found following UV treatment and calculated after SEC analysis: results represent the mean +/- SD of 4 batches; **(c)** volume-weighted profile for the evaluation of size distribution of IFX N and IFX UV, obtained by dynamic light scattering (representative results of one batch).

Aggregates (IFX UV) were generated by submitting native IFX (IFX) N at a concentration of 1 mg/ml to a UV stress (365nm) for 20 hours. IFX N or IFX UV were first analyzed by size exclusion chromatography (SEC). A single peak of monomer was observed for IFX N (**Figure 1a**). For IFX UV, a significant decrease of the monomer peak’s area was observed in comparison to IFX N. The trimer (retention time 6.2 min), dimer (retention time 7.7 min) and monomer (retention time 8.9 min) together with several fragments (retention time 10.7 min, MW∼50 kDa) were also detected by fluorescence detector (FLD) (**Figure 1a**). Our results based on the calculation of the ratio of the relevant peak areas showed that after 20 hours of incubation, 52.25% ± 3.83% of monomeric IFX was left in the aggregated sample and a mixture of 19.27% ± 0.24% dimers and 26.19% ± 3.95 trimers were formed (**Figure 1b**).

These percentages were confirmed by the analysis of IFX UV preparations by Field Flow Fractionation (FFF) combined to a multi-angle light scattering (MALS) detector (**Supplementary Data 1a, b**). Using FFF-MALS, we found a proportion of monomers representing around 50% of the total forms, whereas the dimers (22.4%), trimers and oligomers (24.7%) accounted for the remaining portion.

IFX N and UV solutions were then analyzed by dynamic light scattering (DLS). The size profiles are reported using volume-weighted analysis. At a concentration of 1 mg/ml, average sizes of IFX N corresponded to 17.5 ± 2.4 nm (median 16.9 nm), The aggregated sample resulted in the formation of large particles with average size of 37.5 ± 26.8 nm (median 37.62 nm), confirming the formation of small-sized aggregates during exposure to UV stress. Moreover, no sub-micrometric aggregates (>100nm) could be observed by DLS (**Figure 1c**).

IFX N or UV were subjected to SDS-PAGE. Under non-reducing conditions, IFX N and IFX UV preparations migrated with one major band corresponding to monomer at 150 kDa. In contrast, IFX UV showed multiple bands of varying sizes, ranging from 100 to over 250 kDa, suggesting the presence of multimeric IgG in the sample (**Supplementary Data 1c**). When disulfide bonds were reduced, we observed the separation of the two Ab chains in IFX N, with the heavy chain appearing at 50 kDa. In contrast, while some of the multimeric IgG in IFX UV were disrupted under these conditions, others were detected in the wells (**Supplementary Data 1d**). These findings suggest the presence of covalently bound aggregates in the aggregated samples.

### 2.2 Identification of pre-existing IFX-native and IFX-aggregates responding CD4 T cells

**Figure 2:**
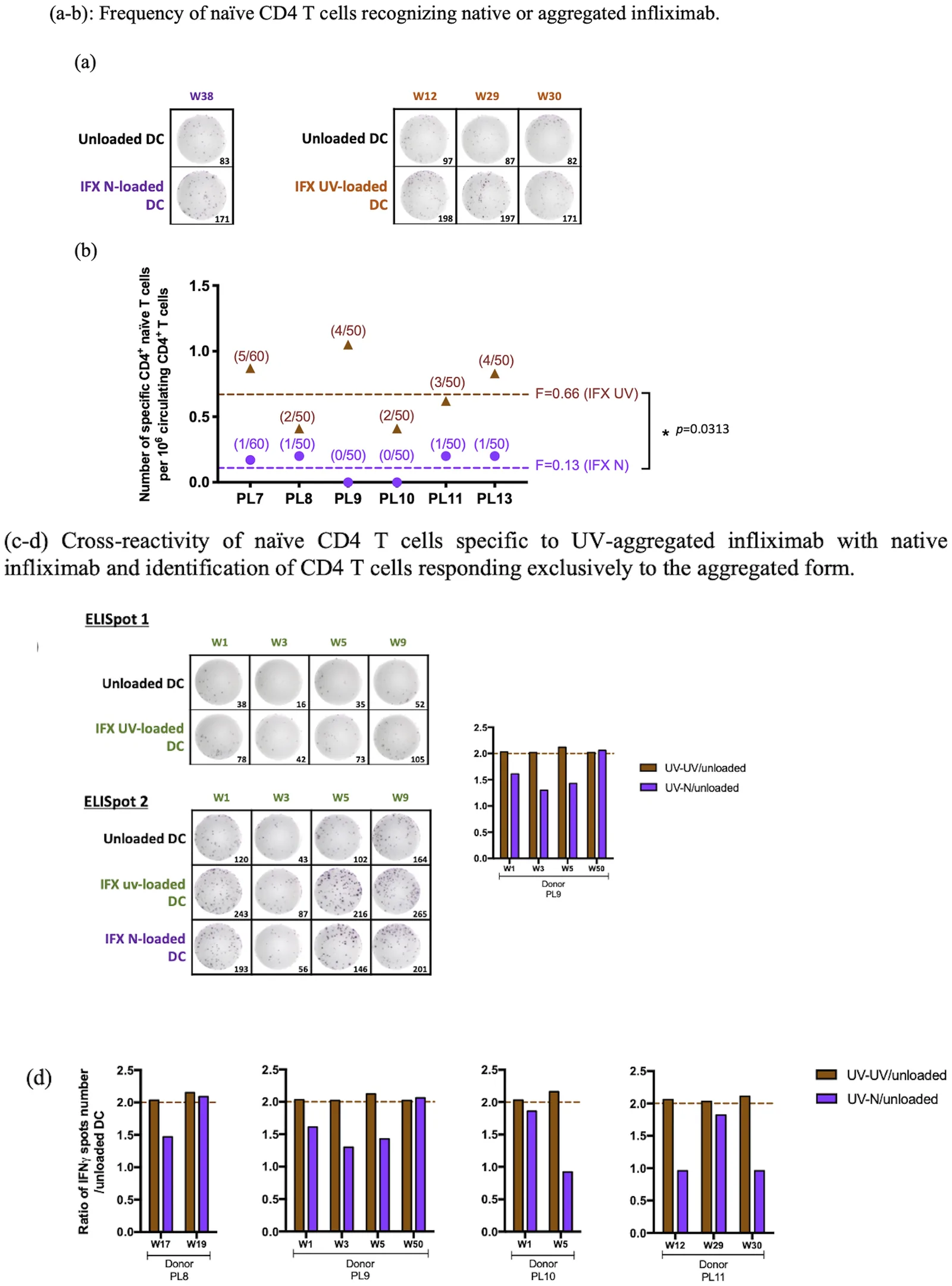
Pre-existence of native and aggregated infliximab specific naïve CD4 T cells and their shared T-cell recognition. **(a, b)** CD4 T cells from healthy donors were repeatedly stimulated *in vitro* with native or aggregated IFX. After 3 weeks of co-culture, CD4 T cell response was analyzed by IFN-γ ELISpot assay. **(a)** IFN-γ, ELISPOT response of naïve CD4+ T cells from donor PL11 stimulated with unloaded DC or DC loaded with IFX N or UV **(b)** Frequency of human naïve CD4+ T cells specific for IFX N or UV. The frequency was calculated for each donor using the Poisson distribution law as described in the Material and Methods section. On the x-axis is represented the donor’s number. For the different donors tested, the number of T-cell lines found specific for IFX N or UV out of the number of T-cell lines tested is indicated in bracket. The statistical significance was assessed using the Wilcoxon matched pairs signed rank test (*p<0.05). **(c, d)** CD4 naïve T cells specific of IFX UV identified in the first ELISpot assay were tested in a second ELISpot assay for their response towards IFX (N) These T cells were stimulated with unloaded moDCs, or moDCs loaded with IFX UV or IFX N **(c)** Representative results shown for donor PL9: images and spot-count for naïve CD4 T cells recognizing IFX UV. IFX UV-specific T cells detected in the first ELISpot assay were stimulated with unloaded moDCs or moDCs loaded IFX N or UV; histogram represents ratio of IFN-γ spots detected in response to IFX UV (brown bars) and IFX N (purple bars) compared to IFN-γ spots detected in response to unloaded moDCs calculated for donor PL9; **(d)** Histograms showing results for donors, PL8, PL9, PL10 and PL11: ratio of IFN-γ spots detected in response to IFX UV (brown bars) and IFX N (purple bars) compared to IFN-γ spots detected in response to unloaded moDCs were calculated for each of the four tested donors.

As the presence of pre-existing naïve CD4+ T cells recognizing protein epitopes is necessary to mount a specific T-cell response, we assessed the response of CD4+ T lymphocytes to IFX N or IFX UV. Naïve CD4+ T cells obtained from six healthy donors were seeded in 96-well plates and stimulated weekly by mature moDCs loaded with IFX N or IFX UV. Each independent T-cell line (T cells present in a single well) was evaluated for its ability to recognize IFX N or UV using an IFN- γ Elispot assay where each T-cell line was stimulated with unloaded DCs, IFX N or UV-loaded DCs. Although all wells were treated under the same condition, antigen specificity of the resulting T-cell lines was different across the wells. For instance, for donor PL11, 1 out of 50 CD4 T-cell lines responded to IFX N while 3 out of 50 CD4 T-cell lines responded to IFX UV (**Figure 2a**; **Supplementary Data 2**). Naïve CD4+ T cells that were able to recognize IFX N or UV were detected for all 6 tested healthy donors. We then used these results to calculate the frequency of preexisting IFX N or IFX UV-recognizing naïve CD4+ T cells for each donor using the Poisson distribution law. We found for each of the tested donor a higher number of responding CD4 T cells for IFX UV compared to IFX N with a mean frequency of 0.13 and 0.66 IFX N or IFX UV-recognizing naïve CD4+ T cells per million of circulating naïve CD4+ T cells respectively (**Figure 2b**). These findings indicate that IFX aggregation enhances T-cell recruitment, potentially by increasing the abundance or diversity of IFX-derived peptides presented by APCs, or by engaging additional T-cell receptor specificities within the naïve repertoire.

### 2.3 Overlapping T-cell responses of IFX aggregate-recognizing naïve T-cells to native IFX

To determine whether naïve CD4+ T cells recognizing IFX aggregates, identified in the first ELISpot assay, were also able to recognize IFX N, we tested in a second ELISpot assay, their capacity to respond to native IFX. We identified 11 naïve CD4-T cell lines recognizing IFX UV in the first ELISpot assay from four different donors (**Figure 2c**). Our results showed that from all donors combined 7 IFX UV-recognizing naïve CD4+ T-cell lines recognized only IFX-UV while 4 were positive for both IFX N and IFX UV. As an example, for donor PL9, 4 out of 50 tested T-cell lines in the first ELISpot assay were specific for IFX UV. The second ELISpot assay showed that only 1 (W50) of the 4 positive cell lines was able to recognize both IFX N and IFX UV (**Figure 2c**). However, the 3 other wells (W1, W3 and W5) were positive only for IFX UV. Results for the other three donors are shown in **Figure 2d**. These results suggested that some peptides presented by moDCs and recognized by CD4+ T cells were common to the two forms of Abs, while others responded only to peptides derived from IFX UV. Conversely, the specificity of IFX N-recognizing naïve CD4+ T cells was also tested. Thus, some T-cell lines were able to recognize both IFX N and IFX UV, which is not surprising considering the presence of residual monomer in the aggregated IFX preparation, that could be sufficient to potentially be recognized by some T clones (data not shown).

### 2.4 Distinct TCR repertoire between IFX N- and IFX UV-specific CD4 T cells

**Figure 3:**
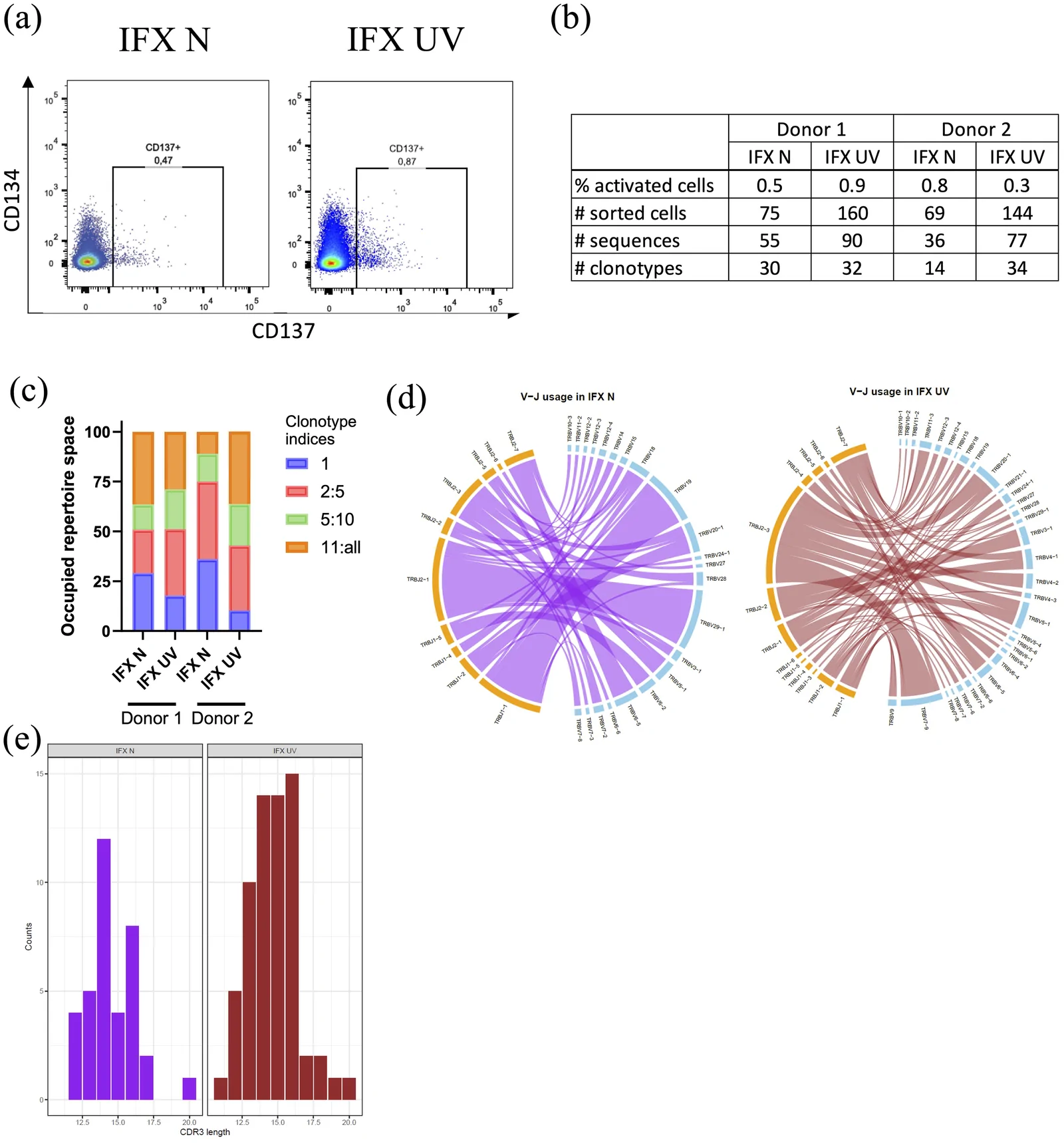
TCR characterization of IFX-specific CD4 T cells. Data were collected from two healthy donors. After 3 weeks of moDC and CD4 T cell co-culture in presence of IFX N or IFX UV, IFX-specific CD4 T cells were sorted by flow cytometry based on their expression of the activation marker CD137 and scTCRseq were proceeded in these cells. **(a)** Dot plots representing CD137 expression on CD4 T cells after IFX N or IFX UV-stimulation. **(b)** Summary table containing information about the frequency of activated cells, number of sorted cells, number of sequences obtained after scTCR sequencing and number of clonotypes for each donor and each condition (IFX N or IFX UV). **(c)** Histrograms representing the distribution of IFX-specific clonotypes as a function of their relative abundance. **(d)** Circos plots representing the V-J gene usage of the TCRβ in the IFX N-specific cells (left panel) and the IFX UV-specific cells (right panel). **(e)** Histograms representing the frequency of clonotypes according to the CDR3β length in IFX N-specific cells (left panel) and the IFX UV-specific cells (right panel).

To further characterize IFX N- and IFX UV-specific CD4 T cells identified previously, we performed single-cell sorting and scTCR-seq of antigen-specific cells from two healthy donors. Using the co-culture assay previously described, IFX N and IFX UV treatment generated 5 and 11 antigen-specific T cell lines, respectively, for the first donor, and 6 and 11 antigen-specific T cell lines, respectively, for the second donor, out of 96 T cell lines tested per antigen. Following identification of these positive T cell lines, IFX-specific CD4^+^ T cells were sorted based on the expression of the activation marker CD137 (**Figure 3a**) and processed for scTCR-seq. A summary of the sorting and sequencing workflow is shown in **Figure 3b**. Higher number of sorted cells, productive sequences and clonotypes were obtained in the IFX UV condition compared to IFX N. Notably, the top five clonotypes accounted for >50% of the specific repertoire across all samples (**Figure 3c**), suggesting substantial expansion of a limited number of clonotypes. Comparison of the TCRβ V-J gene usage revealed distinct repertoire between IFX N- and IFX UV-specific clonotypes: TRBV29–1 paired with TRBJ2–1 was the most frequent V-J combination in IFX N clonotypes while TRBV7–9 paired with TRBJ2–3 predominated in IFX UV clonotypes (**Figure 3d**). Additionally, CDR3β length distribution differed, with a peak at 14 amino acids for IFX N and at 16 amino acids for IFX UV (**Figure 3e**). Together, these results indicated that IFX N and IFX UV recruited different CD4 T cell populations with a distinct TCR repertoire.

### 2.5 Effects of IFX aggregates on moDC phenotype

To study the impact of aggregation on moDCs’ phenotype as a parameter accounting for T-cell activation, we analyzed the expression of surface markers, cytokine and chemokine production by moDCs after 24 hours of stimulation with 200µg/ml of IFX N or IFX UV. We showed that IFX N or IFX UV had no significant effect on the expression of CD83 and CD86 (**Supplementary Data 3a**). Results also showed no significant increases of IL-6, IL-8, IL-12p40, IL-10, CCL-3 and CCL-4 levels compared to the untreated control (**Supplementary Data 3b**). Taken together, these results showed that IFX N or IFX UV do not induce moDCs activation based on surface markers expression and cytokines or chemokines release.

### 2.6 Internalization of native and aggregated IFX by moDC

**Figure 4:**
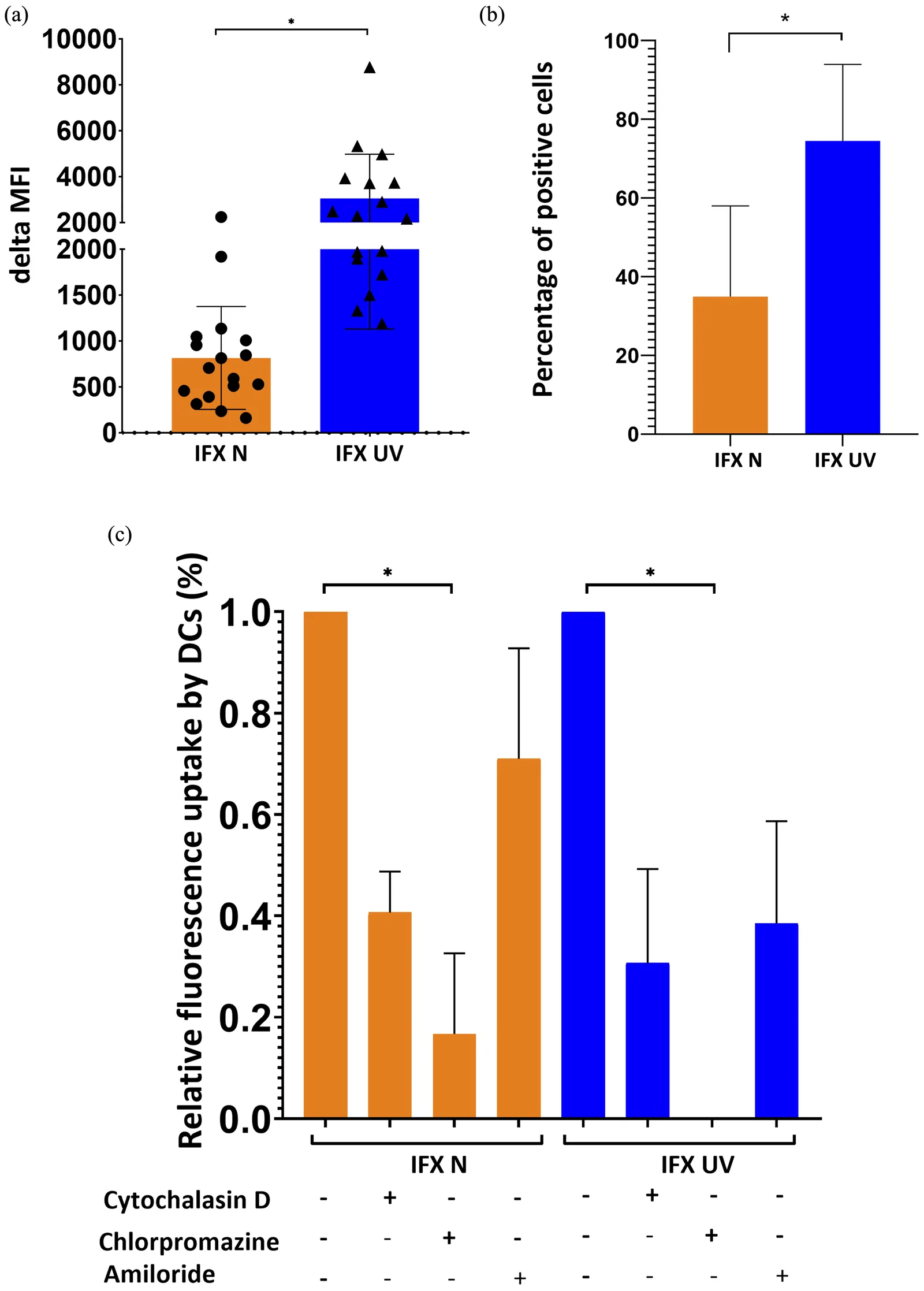
Uptake of native and aggregated infliximab by moDC. moDC were incubated for 2 hours with Alexa Fluor 488 (AF-488) labelled native (N) or aggregated (UV) infliximab (IFX) at 37 °C or 4 °C **(a)** Internalization rate was evaluated by flow cytometry and expressed as delta of the mean fluorescent intensity (MFI), calculated from the difference between the MFI of surface-localized (4°C) and internalized (37°C) IFX N or IFX UV; results are expressed as the mean+/- SD of 18 donors, *p<0,05 Wilcoxon signed-rank test **(b)** percentage of cells internalizing IFX N or IFX UV; mean +/- SD of 18 donors, *p<0,05 Wilcoxon signed-rank test **(c)** moDCs were pre-treated for 1 hour with either cytochalasin D, chlorpromazine or amiloride before incubation with fluorescently labelled IFX N or IFX UV. Results are expressed as relative uptake of IFX N or IFX UV by DCs defined as (37 °C MFIinh-4 °C MFIinh)/(37 °C MFIcondition -4 °C MFIcondition), where ‘‘MFIinh’’ stands for MFI detected in the presence of the inhibitors and “MFI condition” stands for MFI detected in the presence of IFX N or UV (n=3); *p<0,05 Kruskal Wallis followed by Dunn’s multiple comparison test.

Since Abs internalization by APCs is a crucial step for the initiation of a T-cell response, we assessed the uptake of fluorescent IFX N or IFX UV by immature moDCs using flow cytometry. The immature state of moDC was first confirmed by the low expression of CD83 (<2%) and CD86 (<30%). The degree of labelling (DOL) of IFX N and IFX UV labelled with AF-488 dye was found comparable with 3 and 3.4 AF-488 per mole of IFX N and IFX UV respectively. Similar chromatograms (**Supplementary Data 4a, b**) and species proportion (**Supplementary Data 4c**) for both labeled and unlabeled IFX N and IFX UV preparations were obtained by SEC analysis. A small loss of protein due to dialysis was observed. These results suggested that labelling did not have a major influence on proteins structure. Following loading of moDCs with IFX N or IFX UV, uptake was calculated by the difference in the binding signal at 4°C and the residual signal at 37°C. Calculation of fluorescence intensities gave higher rates of internalization for IFX UV compared to IFX N for all tested donors (n=18) after 2 hours of incubation (**Figure 4a**). Moreover, we showed that a higher percentage of positive cells was found for IFX UV in comparison to IFX N with 71% ± 19% and 27% ± 22% positive cells respectively (**Figure 4b**). The cell fluorescence progressively increased along time for both Alexa fluor (AF) 488-conjugated IFX N and AF488-conjugated IFX UV (**Supplementary Data 5**). However, given the limited biological replication (n = 2), these data should be interpreted as qualitative supporting evidence. Taken together, these results showed that IFX UV was taken up more efficiently, as compared to the IFX N.

To better define the pathways involved in the internalization of IFX N or UV, we used a set of inhibitors targeting the main internalization pathways in DCs (macropinocytosis, phagocytosis, and receptor-mediated endocytosis). The internalization rate of IFX N or IFX UV was reduced by the three tested inhibitors albeit with varying magnitude (**Figure 4c**). Phagocytosis of IFX N and IFX UV was inhibited by 66 ± 15% and 75 ± 18%, respectively, by cytochalasin D, a drug that prevents actin polymerization. Amiloride, a potent inhibitor of macropinocytosis, had a slight effect on IFX N’s internalization, with a reduction of up to 32 ± 22%. In contrast, its effect on IFX UV uptake was more pronounced, showing a 67 ± 22% reduction, though this was not statistically significant. Chlorpromazine, an inhibitor of clathrin-coated pit formation, markedly reduced the uptake of both AF-488-labeled IFX N and IFX UV, decreasing IFX N uptake by 90 ± 16% and IFX UV uptake by 100%. The percentage of internalizing cells was slightly different in the presence of cytochalasin D and amiloride while it was significantly reduced for cells pre-treated with chlorpromazine (data not shown). Therefore, these results suggested that both forms of IFX are mostly internalized by moDCs via clathrin-dependent endocytosis.

### 2.7 Receptors implicated in IFX aggregates interaction with moDC

**Figure 5:**
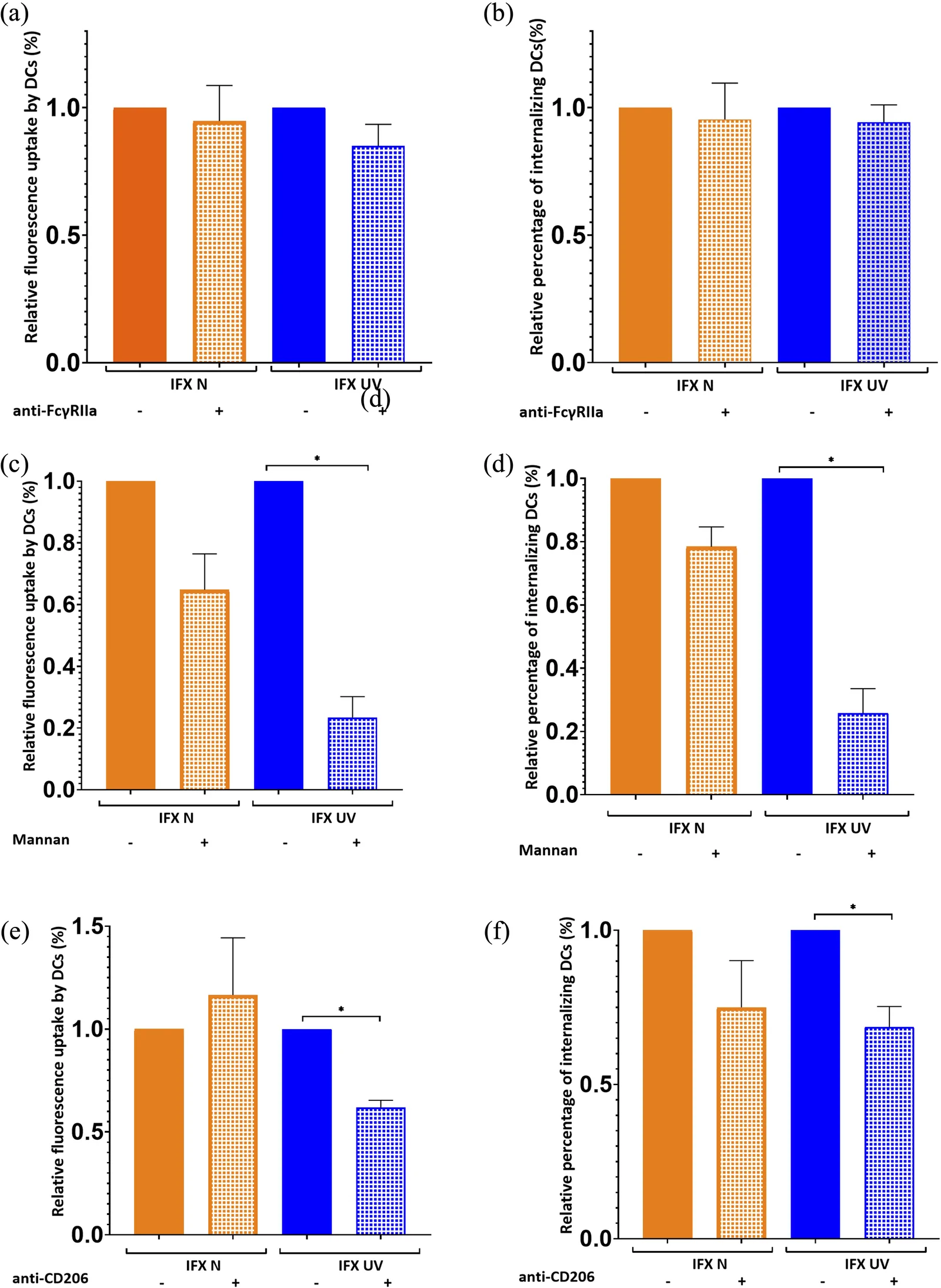
IFX aggregates are internalized through mannose sensitive receptors pathway. **(a–f)** moDC were pre-treated for 1 hour by an anti-FcγRIIa (1µg/ml) (a-b), mannan (2 mg/ml) **(c, d)** or anti-CD206 (10µg/ml) **(e, f)** and then incubated for 2 hours with native (N) or aggregated (UV) infliximab (IFX) labelled with Alexa Fluor 488 (AF-488) at 37 °C or 4 °C and internalization was evaluated by flow cytometry. Results are expressed as relative fluorescence uptake **(a, c, e)** defined as (37 °C MFIinh-4 °C MFIinh)/(37 °C MFIcondition -4 °C MFIcondition), where ‘‘MFIinh’’ stands for MFI detected in the presence of the inhibitors and “MFI condition” stands for MFI detected in the presence of IFX N or IFX. Results are also expressed as relative percentage of internalizing DCs **(b–f)** defined as (37 °C AF488 positive cells inh-4 °C AF488 positive cells inh)/(37 °C AF488 positive cells condition -4 °C AF488 positive cells condition), where ‘‘AF488 positive cells inh’’ stands for percentage of cells positive for AF488 detected in the presence of the inhibitors and “AF488 positive cells condition” stands for percentage of positive cells for AF488 detected in the presence of IFX N or UV (b-d-f); mean +/- SEM of 5 donors. Bars represent relative uptake of IFX N or IFX UV by DCs. Solid bars indicate untreated conditions, whereas patterned bars indicate inhibitor-treated conditions. *p<0,05 Kruskal Wallis followed by Dunn’s multiple comparison test.

Human moDCs are a cellular model known to express various endocytic receptors. In our hands, moDCs expressed Fc receptors (FcRs), particularly Fc gamma receptors (FcγRI and FcγRIIa), as well as C-type lectin receptors (CLR) such as mannose sensitive receptors (DC-SIGN and CD206) (**Supplementary Data 6**). Recognition of Abs by these receptors is dependent on the glycosylation of the Fc fragment (35, 36). Since IFX carries an N-linked glycan on its Fc region (37), we investigated the contribution of these receptors to IFX N or UV endocytosis. To demonstrate that CLR or FcRs mediate the internalization of IFX N or IFX UV, we assessed their internalization in the presence of receptor ligands or recombinant proteins as blocking agents. Pre-treatment of moDC with anti-FcγRIIa Ab, showed that endocytosis of IFX N was not inhibited while IFX UV’s uptake was slightly reduced by 19% (**Figure 5a**). The percentage of internalizing cells was also not significantly reduced after inhibition (**Figure 5b**). We then pre-treated DCs with 2 mg/ml of mannan, a competitive ligand for mannose-sensitive uptake, and evaluated IFX N and IFX UV internalization by flow cytometry as previously described. We found that pre-incubation of moDCs with mannan significantly reduced the internalization rate of IFX UV by 76 ± 14% and the percentage of internalizing cells by 77 ± 17%, whereas IFX N uptake showed a greater variability and was not significantly inhibited (**Figures 5c, d**). These results indicated that mannose-sensitive receptors mediated a significant part of the endocytosis of IFX UV by moDCs. To go further with this observation, we wanted to identify the receptors engaged in this process.

In our hands, moDCs express the macrophage mannose receptor (CD206) and DC-SIGN (CD209), receptors that bind carbohydrates in a calcium-dependent manner (data not shown). CD206 is a transmembrane CLR with C-type lectin domains (CTLDs) responsible for mannose/N-acetylglucosamine/fucose-terminated ligand binding and endocytosis (38). DC-SIGN is a type II transmembrane glycoprotein receptor with an ectodomain consisting of a neck region and a CTLD essential for recognizing mannose-containing structures (39). Blockade of the mannose receptor using a saturating concentration of anti-CD206 IgG (10 µg/ml) resulted in a significant inhibition of the internalization rate of IFX UV by 39 ± 8% and the percentage of internalizing cells by 32 ± 15%. IFX N uptake showed a greater variability and was not significantly inhibited (**Figures 5e, f**). Moreover, we showed that blocking DC-SIGN with a specific antibody did not modify IFX N or UV uptake (**Supplementary Data 7a**). We also showed that the DG-75 DC-SIGN^+^ cell line, despite functional overexpression of DC-SIGN, does not exhibit increased internalization of IFX N or UV compared with the wild-type counterpart. The internalization capacity of DG-75 wild type or DC-SIGN^+^ was addressed using labelled ovalbumin, for which internalization was increased in DC-SIGN^+^ cells compared with the wild-type cells (**Supplementary Data 7b**). As these experiments were performed with only two biological replicates, they are presented as supporting observations that complement the primary dataset.

Taken together, these results support the contribution of the mannose-sensitive receptors, partly through CD206, as a pathway for IFX aggregates internalization.

### 2.8 IFX UV are more efficiently targeted to endo-lysosomal compartments

**Figure 6:**
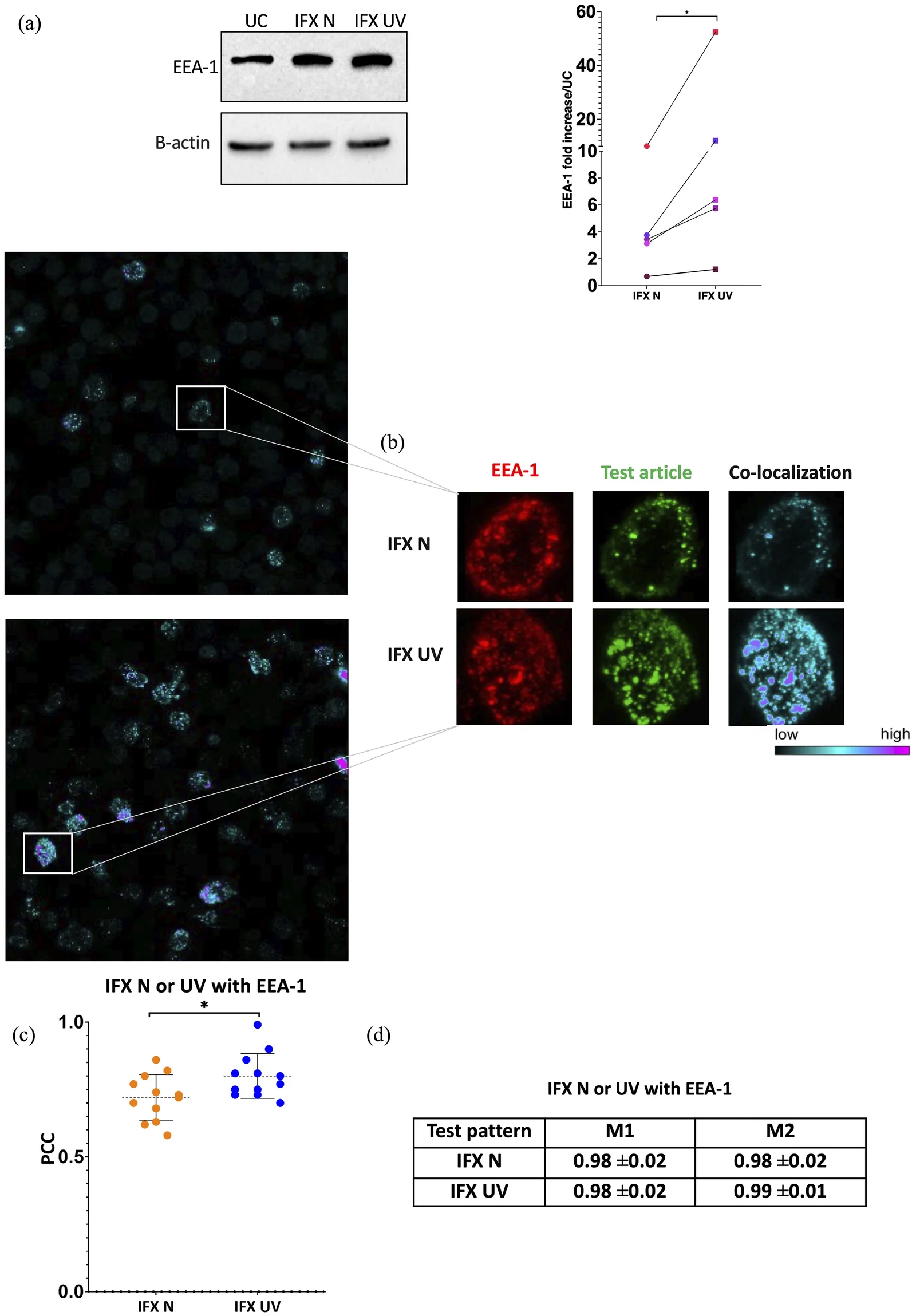
IFX N and IFX UV are routed to endosomal compartments. **(a)** EEA-1 expression following moDC treatment with native (IFX N) or aggregated (IFX UV) infliximab. Immature moDC were treated for 5 min with IFX N or UV. Immunoblotting of whole-cell extracts was used to quantify EEA-1 expression. The results of 6 independent experiments are represented. Bands were quantified using the Image Lab software. Results are expressed as the fold induction, representing the ratio of the normalized intensity of specific bands of treated cells divided by the normalized intensity of bands of untreated cells (UC); *p<0.05 Wilcoxon signed-rank test. **(b–d)** moDCs from healthy donors were incubated with Alexa-Fluor 488-labeled native (IFX N) or UV-treated IFX (IFX UV) (green) at 37 °C for 15 minutes to visualize early endosomal compartments. Cells were fixed, permeabilized, and immunostained with anti-EEA-1 antibodies (red). Fluorescence was detected by laser scanning confocal microscopy at 488 nm (green) and 674 nm (red). The color scale bar beneath each image indicates the degree of colocalization. **(c, d)** Colocalization of IFX N or IFX UV (green) with EEA-1 (red) was quantified using Pearson’s correlation coefficient (PCC) and Manders’ correlation coefficient (MCC). **(c)** Scatter plot of PCC values for IFX N or IFX UV with EEA-1. **(d)** MCC values: M1 represents the proportion of HLA-DR signal overlapping IFX N or IFX UV, and M2 represents the proportion of IFX N or IFX UV signal overlapping EEA-1 Data are presented as mean ± SD (n = 12 cells; N = 4 biological replicates). *p< 0.05, paired Student’s t-test.

Once internalized in DCs, antigens are targeted into early endosomes from which they are sorted to the recycling pathway or rapidly transit to lysosomes to be processed into peptides. EEA-1 is a cytosolic protein that specifically binds to early endosomal membranes. moDCs were treated with IFX N or IFX UV for 5 minutes, prior to cell lysis. Results showed that EEA-1 expression was significantly increased in IFX UV treated cells compared to IFX N-treated moDCs (**Figure 6a**; **Supplementary Data 8**). This observation suggested that EEA-1 was more recruited to the endosome membrane with IFX UV. Then, using confocal microscopy, we addressed the colocalization with endosomal compartments of IFX N and IFX UV. Confocal images showed an extensive co-localization between EEA-1 and punctate IFX N or IFX UV distributed throughout the cytosol (**Figure 6b**; **Supplementary Data 9a**). To provide a comprehensive and quantitative analysis, we applied 2 types of colocalization analysis: Pearson correlation coefficient (PCC) and Mander’s colocalization coefficient. PCC is an indicator of functional relationships while Manders’ coefficients (M1 and M2) were used as quantifiers of spatial overlap between fluorophores. We found a considerable level of overlap (PCC 0.7 **± **0.1 for IFX N and 0.83 ± 0.1 for IFX UV) suggesting a strong colocalization between IFX N and early endosomes. This colocalization was significantly greater for IFX UV. However, there was no difference in the fraction of endosomes containing IFX N and IFX UV (M1 0.98 ± 0.02) and almost all internalized particles were associated with EEA-1 (M2 > 0.95) (**Figures 6c, d**).

**Figure 7:**
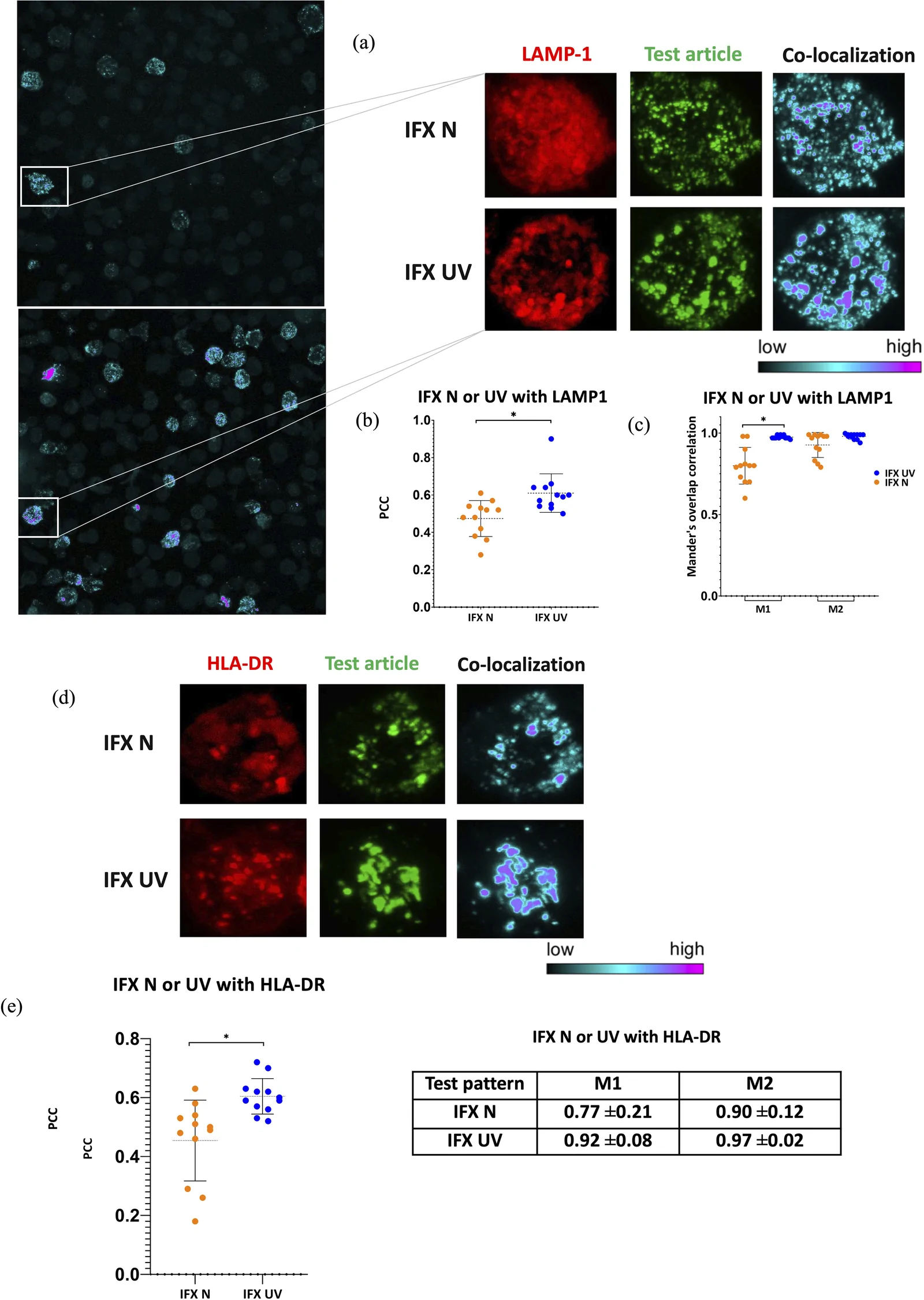
IFX aggregates are more efficiently targeted to lysosomal compartments. **(a)** moDCs from healthy donors were incubated with Alexa-Fluor 488-labeled native (IFX N) or UV-treated IFX (IFX UV) (green) at 37 °C for 60 minutes to visualize late endosomal/lysosomal compartments. Cells were fixed, permeabilized, and immunostained with anti-LAMP-1 antibodies (red). Fluorescence was detected by laser scanning confocal microscopy at 488 nm (green) and 674 nm (red). The color scale bar beneath each image indicates the degree of colocalization. **(b, c)** Colocalization of IFX N or IFX UV (green) with LAMP-1 (red) was quantified using Pearson’s correlation coefficient (PCC) and Manders’ correlation coefficient (MCC). **(b)** Scatter plot of PCC values for IFX N or IFX UV with LAMP-1. **(c)** Scatter plot of MCC values: M1 represents the proportion of LAMP-1 signal overlapping IFX N or IFX UV, and M2 represents the proportion of IFX N or IFX UV signal overlapping LAMP-1. **(d)** moDCs were incubated with Alexa-Fluor 488-labeled IFX N or IFX UV (green) at 37 °C for 120 minutes and immunostained with anti-HLA-DR antibodies (red). Fluorescence was detected by confocal microscopy as described above. The color scale bar indicates the degree of colocalization. **(e, f)** Colocalization of IFX N or IFX UV (green) with HLA-DR (red) was quantified using PCC and MCC. **(e)** Scatter plot of PCC values for IFX N or IFX UV with HLA-DR. **(f)** MCC values: M1 represents the proportion of HLA-DR signal overlapping IFX N or IFX UV, and M2 represents the proportion of IFX N or IFX UV signal overlapping HLA-DR. Data are presented as mean ± SD (n = 12 cells: N = 4 biological replicates). *p< 0.05, paired Student’s t-test.

We then assessed the endo-lysosomal routing of IFX N or IFX UV. moDC were incubated at 37 °C with AF-488 labeled IFX N or IFX UV for 60 min, then labeled for LAMP-1 subcellular marker. Fluorescent IFX N and IFX UV appeared in punctuate structures inside the lysosomes (**Figure 7a**; **Supplementary Data 9b**). PCC values indicate a strong co-localization (PCC: 0.58 ± 0.1) of intracellular IFX UV with lysosomes while around 0.44 ± 0.1 (PCC) of the IFX N were targeted to these compartments (**Figure 7b**). These results indicate that aggregation is associated with increased localization of IFX to lysosomal compartments. On the other hand, all internalized particles were localized within LAMP-1–positive compartments, as indicated by M2 Manders’ coefficient (M2 0.92 ± 0.09 for IFX N and 0.98 ± 0.02 for IFX UV). However, there was a significant difference in the fraction of lysosomes containing IFX N and UV as measured by the M1 Manders’ coefficient. At 2 hours post-internalization, 88% ± 12% of the lysosomes fraction colocalized with IFX N, compared to IFX UV 97% ± 2% for IFX UV (**Figure 7c**).

### 2.9 IFX UV are more efficiently linked to HLA-DR

Binding of antigenic proteins/peptides to MHC-II occurs in the late endosomal/lysosomal MHC class II compartments (MIIC) before cell surface presentation. Therefore, we evaluated the binding of IFX N or UV to MHC class II molecules. moDCs were incubated at 37 °C with AF-488 labeled IFX N or IFX UV for 120 min, then labeled for intracellular HLA-DR. Fluorescent IFX N and IFX UV accumulate in punctate cytoplasmic structures containing HLA-DR molecules (**Figure 7d**; **Supplementary Data 9c**). We showed a strong co-localization (PCC: 0.60 ± 0.06) of intracellular IFX UV with MHC-II molecules compared to 0.43 ± 0.14 (PCC) for IFX N. These results indicate that aggregation is associated with increased binding and accumulation of IFX in HLA-II–positive compartments (**Figure 7e**). Moreover, 77% of HLA-II-containing compartments were positive for IFX N (M1: 0.77 ± 0.21) compared to IFX UV showing a higher M1 coefficient of 0.92 ± 0.08. Notably, nearly all internalized IFX N or UV showed strong association with HLA-DR, with M2 coefficients ≥ 0.90. (**Figure 7f**).

### 2.10 Mannose-dependent internalization of IFX aggregates influence to T-cell proliferation

**Figure 8:**
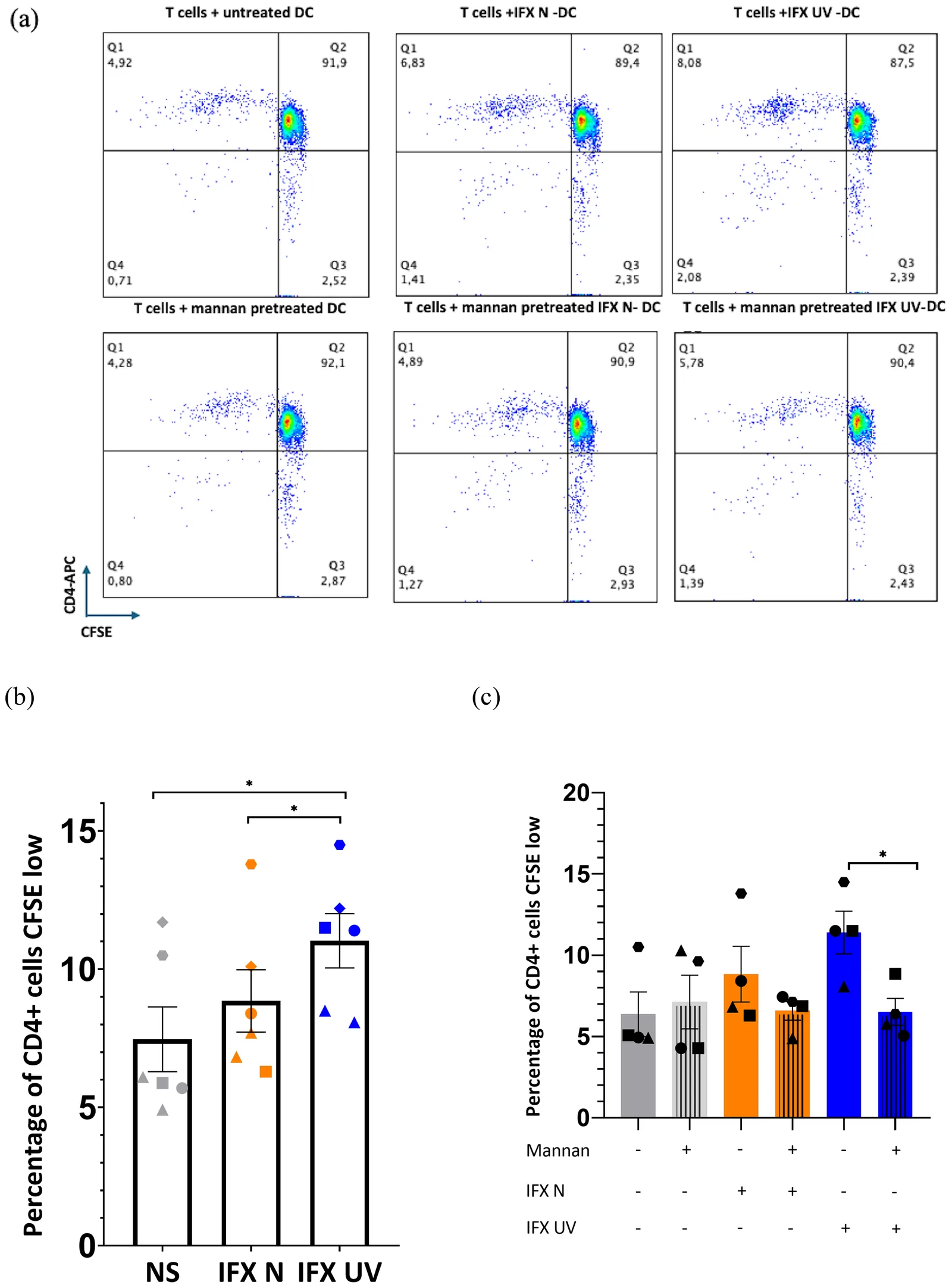
Mannose-dependent endocytosis of IFX UV influence T-cell proliferation. After purification, T cells were labeled with CFSE and incubated with non-stimulated (NS) moDCs or moDCs previously stimulated with native (IFX N) or UV-aggregated (IFX UV) at 200µg/ml at a ratio of 1:5 (T cell/DC). Cocultures were then incubated for 5 days, and proliferation was analyzed by flow cytometry. **(a)** Results are presented as dot plot of one representative experiment, representing the percentage of CD4+ CFSE low cells. **(b, c)** Percentage of CD4+ CFSE low T cells in response to stimulated moDCs with IFX N or UV pretreated or not with mannan (2mg/ml). Results for lymphocyte proliferation and cytokine production are expressed as the mean ± SD of four or six independent experiments. *p<0.05 by Wilcoxon signed-rank test or Kruskal Wallis followed by Dunn’s multiple comparison test.

To evaluate the functional consequences of mannose-dependent internalization of IFX aggregates by moDCs, we investigated the impact of internalization on T-cell activation in an autologous setting. We treated moDCs for 24 hours with IFX N or IFX UV and then co-cultured them with autologous CD4+ T cells loaded with CFSE at a ratio of 1 moDC for 5 CD4+ T cells. CD4+ T cell proliferation was assessed on day 5 as the percentage of CFSE^low^ CD4+ T cells. In a representative experiment (**Figure 8a**), 4,9% of CD4 T cells were CFSE^low^ T cells when incubated with untreated moDCs 6,8% with IFX N, and 8.1% in the presence of IFX UV. While stimulation with IFX N did not alter the capacity of moDCs to induce T cell proliferation compared with unstimulated moDCs, the percentage of CFSE^low^ CD4+ T cells was increased in the presence of moDCs stimulated with IFX aggregates compared with moDCs stimulated with native IFX across all six donors tested. These results suggested again that IFX aggregates are capable of inducing a higher T cell proliferative response compared to IFX N (**Figure 8b**). Moreover, blocking mannose-sensitive receptors by preincubation of DC with mannan before the addition of IFX aggregates, resulted in a significant reduction of the proliferation of T cells (**Figure 8c**). In contrast, no such effect was observed for IFX N (**Figures 8a, c**; representative experiment). Mannan also didn’t prevent the activation of T cell by unstimulated DC (data not shown). These data showed that inhibiting mannose-sensitive endocytosis of IFX aggregates by DCs alter T-cell proliferation.

## 3 Discussion

Aggregation of therapeutic Abs has been associated with increased immunogenicity (40–42). This phenomenon has been reviewed extensively: multivalent aggregate structures can trigger antibody responses independently of T-cell help, notably through cross-linking of B-cell receptors, which both activates B cells and enhances antigen presentation to MHC class II, thereby promoting T-cell help for antibody responses (43). Most of the *in vitro* studies evaluating the role of aggregation on the activation of immune cells such as DCs and CD4 T cells focused on visible and subvisible aggregates (29, 42, 44). This study explores the role of nano-sized aggregates of IFX generated after UV stress in the initiation of an immunogenic T-cell response. Although these conditions do not fully reflect actual storage, handling, and transportation conditions (45, 46), the resulting aggregates are comparable in size to those reported at low levels in commercial antibody products, including species formed under stress conditions such as light exposure or transportation (25, 47, 48). The use of controlled stress conditions enables the isolation and characterization of specific aggregate populations that are otherwise difficult to study in complex formulations (16, 49, 50).

IFX aggregates were characterized using three using three orthogonal methods: SEC, FFF-MALS, and DLS, for covering a complete range (1 nm – 10 μm) of particle sizes. These analyses revealed that IFX UV preparations contain a mixture of 53.4% residual monomeric IFX, 21.1% dimers, and 24.2% oligomers, and confirmed the absence of subvisible and visible aggregates. SDS-PAGE experiments showed that some aggregates were covalently bound. These results were expected and in line with the literature showing that UV exposure can result in the formation of covalent bonding by oxidating Abs (46). Moreover, a mix of monomers and aggregates represent a likely situation.

The preexistence of a CD4 T-cell repertoire in healthy donors, recognizing therapeutic Abs has been well documented (51, 52). Using a co-culture model, we found a higher number of T-cell lines responding to IFX UV for each healthy donor tested compared to those responding to IFX N. This original result suggested that aggregation of IFX made it more able to prime and to recruit naïve T-cells compared to IFX N. Interestingly, most IFX aggregates-activated T cells exclusively recognized peptides derived from aggregates with some responding to both aggregated and native forms of IFX as we previously found with heat-stressed aggregates (30). Rombach-Riegraf et al. showed that loading of moDCs with stir-stress aggregated rituximab led to an increase of Ab-specific peptides associated with MHC-II molecules (28). Our results suggested that aggregation could alter the pattern of antigen-derived MHC class II associated peptides presented by DCs. Either more peptides are presented resulting in the recruitment of T-cells with a lower avidity TCR or aggregates generate more diversity of presented peptides, leading to an increased number of responding T-cells.

To further investigate whether this enhanced response was associated with changes in T-cell clonality, we performed TCR sequencing after sorting CD4 T cells based on expression of the activation marker (CD137). We showed that IFX N- and IFX UV-specific CD4 T cells displayed distinct TCR repertoires. Although our analysis was limited by the small number of donors and antigen-specific cells subjected to scTCR-seq, we consistently observed two different repertoires profiles. IFX UV stimulation yielded a higher number of clonotypes with greater diversity (broader V–J gene usage) and a distinct CDR3β length distribution compared with IFX N stimulation. Further studies will be required to expand the repertoire analysis and to link individual TCRs to their cognate epitopes to determine whether IFX N- and IFX UV-specific TCRs recognize the same or distinct regions of IFX. The low frequency of therapeutic Ab-specific CD4 T cells in healthy donors (52, 53) limits their in-depth characterization, particularly with respect to their TCR repertoires. Cassotta et al. identified natalizumab-specific CD4 T cells in two ADA+ patients, characterized the TCR Vβ of 5 and 7 clonotypes for each patient, respectively, as well as their cognate epitopes and MHC restriction (54). Together, these results provide insight into how aggregation of IFX can shape T cell repertoire diversity and immune recognition.

We then addressed the question whereas the higher T-cell response to aggregates could be a consequence of a higher internalization rate by DCs (31, 33, 55, 56). Recently, Wen et al. established a correlation between a higher uptake and proteolytic processing, the initiation of a specific T-cell response and clinical immunogenicity (33). Other studies also showed that immunogenic monoclonal Abs are internalized and processed more efficiently than low or non-immunogenic mAbs (32, 57). We first studied the uptake of IFX N or IFX UV by flow cytometry, by directly labeling both preparations with AF-488. Both labeled preparations had a similar DOL and similar elution profiles from SEC in comparison to the unlabeled form. Same type of observations has been reported elsewhere and showed that labelling of IgG with low amounts of fluorescent probe, does not have a major influence on molecules’ physico-chemical properties (58). Our results showed a higher rate of internalization in DCs (fluorescence intensity and percentage of internalizing cells) for IFX UV in comparison to IFX N for each donor tested. Ahmadi et al. evidenced a more efficient uptake by moDC of stir-stressed rituximab with a wide range of sub-micron and micron-sized aggregates in comparison to an unstressed preparation (29). It is known that intrinsic characteristics of antigens such as charge or hydrophobicity could impact their uptake by DCs (14, 31, 59). Exposure of Abs to light stress induces charge modification (60) which could partially explain our results.

Since endocytosis routes in APC can determine antigen presentation and, consequently, the instructions given to T cells (61, 62), we evaluated the internalization pathways of IFX N or IFX UV in moDCs. Proteins are generally internalized through either macropinocytosis, clathrin-mediated endocytosis or phagocytosis (63). Using well-described pharmacological inhibitors of these three main pathways, we demonstrated that both IFX N and IFX UV uptakes were significantly reduced by chlorpromazine. Chlorpromazine has been largely described as a clathrin/receptor mediated endocytosis inhibitor with still some questions on its absolute specificity for this pathway. Its primary mechanism involves the redistribution of clathrin and adaptor protein 2 (AP-2) from the plasma membrane to intracellular compartments, thereby preventing the formation of functional clathrin-coated pits and reducing receptor-mediated internalization (14, 64). Internalization of IFX N and UV through receptor-mediated endocytosis is not surprising considering their size (10 nm for IFX N and between 10–100 nm for IFX UV) and since it is known that a size threshold determines the endocytic pathway. Aggregated particles larger than 0.5 μm are more prone to be internalized by phagocytosis or micropinocytosis (64).

Clathrin-mediated endocytosis requires the binding of external ligands to a specific receptor on DCs. Indeed, the glycosylation patterns typically present on the Fc region of Abs can influence their interactions with endocytic receptors, particularly FcγRs and CLR, which recognize carbohydrate motifs. Our results showed that, FcγRIIa, the primary FcγR expressed by moDCs, was only slightly involved in the uptake of IFX UV by moDCs. Our group previously demonstrated that the FcγRIIa-Syk axis was the main pathway mobilized in DC activation by micrometric heat stress-induced IFX aggregates and the subsequent T cell response (27). Aoyama et al. demonstrated that the internalization of Ab drug conjugates aggregates was FcγR-dependent (65). Jin et al, showed that internalization by human monocytes and granulocytes of avelumab (anti-PD-L1) was mainly FcγR and PD-L1 dependent (13). Our results strongly suggested that, depending on the size and the type of aggregates, the engaged receptors might differ.

Other receptors, including CLR, may contribute via clathrin-mediated pathways. Supporting this, a recent study demonstrated that interaction with the mannose receptor, a CLR, depends on the high-mannose content of the Ab but is independent of its format or sequence (66). Consistent with these findings, we showed that exposed mannosylation on IFX, reported to account for approximately 5.5% of the N-glycan population (67), is relevant for its interaction with moDCs. Indeed, we observed a 76% reduction of IFX UV internalization rate after blocking mannose-sensitive receptors with mannan, providing consistent evidence that these receptors play a substantial role in the uptake of IFX aggregates for the uptake of IFX aggregates. The inhibition of IFX UV endocytosis was less marked with anti-CD206 IgG than in the case of mannan. One possible explanation is that the multivalent structure of mannan allows it to engage multiple carbohydrate recognition domains (CRDs) on CD206 while simultaneously interfering with other mannose-binding C-type lectin receptors. By comparison, the monoclonal anti-CD206 antibody targets a single epitope and may therefore achieve a lower degree of functional blockade. The finding of a residual IFX UV uptake despite the disruption of the mannose-sensitive pathway indicates also the involvement of alternative endocytic processes for IFX UV that remain to be identified. Importantly, immune complex formation between infliximab and TNF-α, including binding to transmembrane TNF, has been widely recognized as a central mechanism driving cellular uptake. Deora et al. showed that the formation of anti-TNF/transmembrane TNF complexes resulted in their rapid internalization by moDCs following LPS stimulation (68) and *in vivo* studies further support a crucial role for immune complexes in the immunization against anti–TNF-α Abs (69, 70). Taken together, these studies established immune complex formation as a key and well-accepted pathway governing the cellular uptake and immunogenicity of anti-TNF biologics such as IFX. In this study, we suggest that glycosylation-dependent endocytic mechanisms may also contribute for the uptake of aggregated antibodies, potentially extending beyond immune complex–mediated pathways. Glycosylation may provide an additional route for antigen processing and presentation in the context of biopharmaceutical immunogenicity and aggregation. Wolf et al. demonstrated that Fc glycosylation of rituximab was critical for its recognition by DC with the glycosylated Ab binding efficiently to the cells, whereas the aglycosylated form showed markedly reduced binding (15).

Once internalized by DCs, antigens are first trafficked to early endosomes, marked by EEA-1, where they are sorted either for recycling or directed toward lysosomes for degradation into peptides. Antigen processing and MHC class II loading occur in late endosomal/lysosomal compartments (LAMP1^+^), followed by peptide loading onto MHC-II molecules in MIIC (HLA-DR^+^) (63, 71, 72). Using confocal microscopy, we analyzed the co-localization with EEA-1, LAMP-1, and HLA-DR to compare the intracellular distribution of IFX N and UV across the endosomal–lysosomal pathway leading to MHC-II presentation. We found a higher recruitment of IFX UV compared to IFX N to the endosomal membrane after 5 min and a higher colocalization of IFX UV within these sorting compartments (PCC parameter). Aggregation was associated with significantly increased localization of IFX UV to lysosomal compartments, consistent with enhanced uptake and delivery. These results are consistent with another study showing that mannosylation directs the trafficking of Abs to late endosomes and lysosomes (15).

To determine whether increased uptake led to greater pathway engagement, we examined their localization within MHC-II compartments in moDCs. We observed a higher colocalization with HLA-DR within MHC-II in comparison to IFX N, consistent with increased trafficking to antigen-processing compartments. These findings align with prior observations by Ahmadi et al., who reported enhanced uptake of rituximab aggregates by moDCs and their accumulation within MHC-II molecules, consistent with their role in antigen presentation (29).

We then assessed the impact of mannose-mediated endocytosis on T-cell response and, we found that stimulation of moDCs with IFX UV induced a slightly higher T-cell response compared to IFX N. This slight difference was consistent with observations in other antigen-driven systems, where a minority (~1–11%) of proliferating CD4^+^ T cells are antigen-specific in CFSE-based assays (73). Indeed, precursor frequencies of naïve CD4^+^ T cells specific for IFX N or UV are extremely low. In comparison to robust memory responses, proliferative responses are often very limited in systems where antigen-specific T cells are rare; for example, in HIV-1–infected, viremic patients, antigen-specific CD4^+^ T-cell proliferation can be as low as ~0.2–0.5% of the total CD4^+^ T-cell population following antigenic stimulation (74). Inhibition of IFX N or UV’s uptake with mannan resulted in a significative alteration of IFX-UV-induced T-cell proliferation which was not the case for IFX N. Studies have shown that mannosylated antigens which are endocytosed more efficiently by DCs resulted in an enhanced T cell response and mannosylation has been demonstrated to be an effective approach for potentiating antigen immunogenicity (60, 75).

In this study, we highlighted that nanosized IFX aggregates are more efficiently internalized by DCs via mannose-dependent endocytosis, leading to increased routing into endo-lysosomal compartments. Consistent with a role for this pathway in antigen processing, inhibition of mannose-sensitive receptors reduced the T-cell response to aggregates. While peptide generation and MHC class II presentation were not directly assessed in this study, the increased routing to endo-lysosomal compartments together with the mannan-sensitive T-cell activation are compatible with a contribution of this pathway to antigen processing and presentation, ultimately shaping the observed T-cell response to aggregates. The size of the aggregates remained comparable to those reported in clinical contexts, and the use of an enriched aggregate system allowed them to evaluate their uptake pathways and T-cell activation potential. It is therefore possible that at lower aggregate levels the relative contribution of individual pathways may vary. However, the present model provides a controlled framework to elucidate the role of nanosized aggregates in dendritic cell uptake and T-cell activation. Post-production and handling studies indicate that monoclonal Ab products remain predominantly monomeric under storage and stress conditions, with only limited formation of aggregates (76, 77), suggesting that nanosized aggregates, although present at low levels, may still contribute to functional immune interactions.

### 3.1 Strengths and limitations

A key strength of this study is the integration of physicochemical aggregate characterization with functional immunological readouts, enabling mechanistic insights into DC uptake and T-cell activation beyond descriptive analyses, further supported by the use of human primary cells and an antigen-specific TCR readout. Limitations include aggregate concentrations exceeding those typically encountered in clinical practice — findings should therefore be interpreted as mechanistic proof-of-concept — and an enriched aggregate system that may not fully capture real-world aggregate heterogeneity.

### 3.2 Future directions

Dose-response experiments across a range of aggregate levels would help define activation thresholds, and comparative studies using aggregates generated under different stress conditions — mechanical, thermal, oxidative — would clarify whether aggregate morphology, beyond size and concentration, modulates immune processing. Moreover, performing MAPPs (MHC-associated peptide proteomics) assay could allow to identify peptides associated with aggregates uptake.

## 4 Materials and methods

### 4.1 Samples preparation

Lyophilized IFX (Remicade^®^) was commercially available and reconstituted to a final concentration of 1mg/ml with sterile water. Samples were subjected to UV stress at 365 nm for 20 h (25 J/cm²) using a Vilber Lourmat™ Bio-Link 365 UV crosslinker to generate aggregates. IFX N or UV were aliquoted and conserved at −80°C until use. Aggregates stability was checked by size measurements using SEC and DLS after freezing and thawing, and no modifications were recorded upon time. Endotoxin was quantified for each native or aggregated product by using a colorimetric assay (L00350; Genscript), according to the manufacturer’s instructions. This assay kit can detect endotoxin concentration in the range of 0.01–1 endotoxin units/ml. Levels of endotoxin were considered acceptable and under the limit of 0.25 endotoxin unit/ml. For each new batch of IFX, endotoxin levels were measured prior to experiments, ensuring that observed cellular responses were not attributable to endotoxin contamination.

### 4.2 Characterization of native and UV-stressed infliximab

#### 4.2.1 Size exclusion chromatography

SEC–Laser induced fluorescence experiments were carried out using an Agilent 1260 UPLC, and a 1260 LIF Detector (Agilent Technologies). An Agilent Bio SEC-3 column (3 mm, 300 A°, 4.6 3–300 mm) was employed (temperature 25 °C). The mobile phase (50 mM sodium phosphate, 150 mM KCl, 10% isopropanol [pH 6.8]) was isocratically pumped at 300 µl/min. LIF detection was monitored by a fluorescence detector (lex = 280 nm and lem = 350 nm). Sodium phosphate (424370025, Thermo Fisher Scientific) and potassium chloride were purchased from Thermo Fisher Scientific and from EUROMEDEX (P017, EUROMEDEX) respectively. HPLC grade isopropanol was obtained from Merck (34863, Merck). The FLD chromatograms were registered at an excitation wavelength of 280 nm and an emission at 340 nm. Relative levels of aggregation were estimated from 280 nm absorbance chromatograms using the peak area compared to the sum of all the peak areas corresponding to the different forms of the monoclonal Ab.

#### 4.2.2 FFF-MALS

Experiments were performed using an Thermo Scientific Ultimate 30 0–0 HPLC System coupled with the Eclipse AF4 (Wyatt Technology). A multi-detection system including a UV-visible wavelength Ultimate 3000 RS detector, a MALS, Dawn Heleos II detector and a dRI, Optilab T-Rex (Wyatt Technology) was coupled with the AF4 system. UV-visible detector was set at λ= 280 nm, MALS-Qels lamp at λ= 663.8 nm and dRI lamp at λ= 658nm with cell thermostated at 25 °C. Chromeleon 6.8 software was used to control autosampler, pump and Eclipse flows. Acquisition of UV (λ= 280 nm), MALS-Qels and dRI data was performed by the Astra 6.1 software. Performance of the AF4 system for separating and determining molar mass was checked with bovine serum albumin (BSA) solution (2 mg/ml, w/v). Mobile phase used was composed of 0,01M monopotassium phosphate, 0,0027M potassium chloride and 0,137M sodium chloride.

#### 4.2.3 Dynamic light scattering

The size distribution of native and aggregated IFX was determined at 25 °C by DLS using a Zetasizer Nano ZS90 (Malvern Instruments) operating at a fixed scattering angle of 90° and equipped with a laser source of wavelength of 633 nm. Measurements were performed in disposable microcuvettes with four optical faces (Eppendorf UVette) containing an appropriate volume of sample prepared as above or diluted in NaCl to a concentration of 1.0 mg/ml. The size profile was obtained from the volume-weighted distribution, and the hydrodynamic diameter values were calculated using the Stokes–Einstein equation, assuming a spherical shape of the particles, and corresponded to the median diameter derived from the cumulative distribution curve. The polydispersity index was deduced from cumulant analysis. Values are given as mean values of three independent measurements.

### 4.3 Aggregates analysis with polyacrylamide gel electrophoresis

IFX N or UV in sodium dodecyl sulfate (SDS) sample buffer were subjected to electrophoresis under reducing and non-reducing conditions in 10% SDS-PAGE gel. Protein bands were detected with Coomassie blue G-250 staining (1610436, Biorad).

### 4.4 Generation of human monocyte-derived dendritic cells

Human PBMC were purified from buffy coats directly obtained from Etablissement Français du Sang (France). Healthy donors gave their written consent for the use of blood donation for research purposes. Monocytes were isolated from the mononuclear fraction by magnetic positive selection with MidiMACS separation columns and anti-CD14 antibodies coated on magnetic beads (130-050-201, Miltenyi Biotech). Purified monocytes were cultured at 1 x 10^6^ cells/ml in the presence of GM-CSF (550 U/ml) (130-093-868, Miltenyi Biotech) and IL-4 (550U/ML) (130-093-921, Miltenyi Biotech, Bergish Glaadbach, Germany) in RPMI-1640 25mM HEPES GlutaMAX™ (72400047, Thermo Fisher Scientific) supplemented with 10% fetal calf serum (FCS) (A5256701, Thermo Fischer Scientific) 1% Penicillin/Streptomycin (15140122, Thermo Fischer Scientific) 1% sodium pyruvate (11360070, Thermo Fischer Scientific), at 37°C in humidified air containing 5% CO2. Within 4 days, monocytes differentiated into moDCs, and immature phenotype was controlled by flow cytometry (FACS Attune NxT^®^ acoustic focusing cytometer, ThermoFisher Scientific) using anti-CD83 (<2%) (130-110-503, Miltenyi Biotech), anti-CD86 (<30%) (130-116-162, Miltenyi Biotech), anti-CD1a (>70%) (130-111-873, Miltenyi Biotech), and anti-DC-SIGN (>90%) antibodies (130-124-257, Miltenyi Biotech).

### 4.5 Flow cytometry analysis

On day 4, immature moDCs were stimulated with 200 µg/ml of IFX N or UV and incubated at 37 °C for 24 hours. After 24 hours of treatment, moDCs viability and phenotype were analyzed by flow cytometry. Cell viability was assessed in preliminary experiments by propidium iodide (P1304MP, Invitrogen) staining used at a final concentration of 625 ng/ml on a small fraction of moDC cultures. Cell viability after treatment was never<75%. For the phenotypic analysis, the surface labeling procedure was as follows: 2 x10^5^ cells/ml were washed in cold PBS supplemented with 0.5% BSA and stained with mAbs in the dark on ice for 20 min. The following mAbs were used: allophycocyanin-labeled mouse anti-human CD86 (555660; BD Biosciences) and PE-labeled mouse anti-human CD83 (556855; BD Biosciences) (**Supplementary Data 6**). Appropriate isotype controls (BD Biosciences) were used at the same concentration to determine nonspecific staining. Cells were analyzed on an Attune Nxt (Invitrogen) using FlowJo software (version 10; FlowJo), and we used a gating strategy to exclude dead cells, based on the forward scatter/side scatter criteria. The data acquisitions were performed on a minimum of 10,000 living single cells. Results were expressed as the relative fluorescence intensity (RFI), using the corrected mean fluorescence intensity (cMFI), as follows: cMFI = MFI - MFI of isotype control; RFI = cMFI of treated cells/cMFI of untreated cells (UC).

### 4.6 Autologous co-cultures of moDC and naïve CD4 T cells

Naïve CD4+ T cells were isolated from PBMCs by negative selection using specific microbeads (130-094-131, Miltenyi Biotec). Percentage of CD4+CD45RA+CCR7+ T-cells (>90%) was evaluated using flow cytometry (Attune Nxt, Invitrogen). For T-cell stimulation, DCs were loaded with N or aggregated IFX (200µg/ml) for 8 hours in the presence of 1 μg/mL of LPS (L6529, Sigma-Aldrich). DCs were then washed, and 104 cells were added to 105 autologous naïve CD4+ T cells per round-bottom microwell containing 200μl Iscove’s modified Dulbecco’s medium (IMDM) (12440053, Thermo Fischer Scientific) supplemented with 10% human AB serum (H4522, Sigma), 11–000 U/mL of rh-IL-6 (206-IL, R&D Systems) and 10 ng/mL rh-IL-12 (219-IL, R&D Systems) T cells were restimulated on days 7, 14, and 21, with autologous DCs loaded as described above with rh-IL-2 (10 U/ml) (202-IL, R&D Systems) and rh-IL-7 (5 ng/ml) (207-IL, R&D Systems). For each donor, 50 to 60 individual T-cell lines (10^5^ cells/well) were generated per treatment condition, depending on cell availability. Six donors were tested; the number of T-cell lines analyzed per donor and condition is indicated in brackets in **Figure 2b**. Each line was tested in a single well.

### 4.7 IFN-γ ELISPOT assay

Specificity of the T-cell lines was investigated using IFN-γ ELISPOT assay (3420-2APT-2, MabTech) at day 28. T-cell stimulation with KLH (Keyhole Limpet Hemocyanin; Thermo Fisher Scientific) was used as a positive control. The number of spots was determined using the AID ELISpot Reader system (AID). Unloaded moDCs were used as control. T-cell lines were considered specific for IFX N or UV when the spot count was over 25 spots and at least two-fold higher for IFX N or UV -loaded moDCs compared to unloaded moDCs. Antigen-specific naïve CD4 T-cell frequency was estimated using the Poisson distribution according to the following formula: Frequency = -Ln (Negative wells/Total wells tested)/(Naïve CD4 T cells per well).

### 4.8 Purification of IFX N or UV-specific T cells by cytometry

Antigen-specific T cell lines identified as positive by ELISpot assay were pooled together on day 21, after 24 h of rest in IMDM medium in the absence of cytokine stimuli. The pooled T cell lines were re-stimulated overnight with autologous DCs alone (control) or with DCs previously loaded with either 200µg/ml of IFX N or IFX UV. After 24 h of stimulation, cells were harvested in cell staining buffer (phosphate-buffered saline/EDTA 2 mM/bovine serum albumin 0.5%) and then stained for CD4 (130-114-534, Miltenyi Biotec) and CD137 (4-1BB) (309810, Biolegend). For each condition, the CD4+CD137+ population was sorted by flow cytometry (FACSARIA III; Becton Dickinson) in 96-well plates with 3µL of lysis buffer (5% PEG8000, 0.1% Triton X-100, 0.5U/µL RNAse Inhibitor, 0.5µM of oligodT).

### 4.9 Single cell T cell receptor library preparation and sequencing

Reverse transcription, PCR pre-amplification and quality control were adapted from the Smart-seq3 protocol (78). Briefly, reverse transcription was performed using TSO at 42 °C for 90 min followed by 10 cycles of 50 °C for 2 min and 42 °C for 2 min and terminated at 85 °C for 5 min. PCR pre-amplification was performed using barcoded forward primer with the following PCR cycle conditions: 98 °C for 3 min, 20 cycles of 98 °C for 20 s, 65 °C for 30 s, and 72 °C for 6 min; and a final elongation at 72 °C for 5 min. After PCR pre-amplification, wells from the same column were pooled, purified with AMPure XP beads (A63882, Beckman Coulter) and library size distributions were checked on Agilent Bioanalyzer. cDNA concentration was quantified using the Qubit dsDNA HS assay kit (Q32851, Thermo Fisher Scientific). cDNA was then used in two consecutives PCR steps: a first PCR using reverse primers specific to TRAC and TRBC, followed by a second PCR using custom Nextera index primers. Samples were then pooled and purified with AMPure XP beads. Libraries were sequenced at 150-bp paired-end on a iSeq 100 instrument (Illumina).

### 4.10 Analysis of the single cell TCR-sequences repertoire

TCR sequences were analyzed using R, MiXCR (79, 80) and vdjtools for gene alignment (https://vdj.online/). Clonotypes were defined as unique paired combinations of TCR α and β chain sequences.

### 4.11 Labelling of native and UV-stressed infliximab

For preparation of fluorescently labelled IFX N or UV, Alexa Fluor (AF) 488^®^ tetrafluorophenyl ester (A37570, Thermo Fischer Scientific) was obtained from Invitrogen. Both IFX N or UV were labelled with AF 488 by reaction on the amino group of their lysine residues. A pH of 7.4 was chosen for the labelling buffer to achieve selective labelling of the amine termini. The IFX N-AF488 or IFX UV-AF488 were dialyzed back to Phosphate Buffered Saline (PBS) using a Slide-A-Lyzer^®^ G2 (87729, Thermo Fisher Scientific) with a 10 kDa molecular weight cut-off membrane to remove excess dye. The degree of labelling (DOL) was calculated by measuring the absorbance at 280nm and 488nm on a Nanodrop (ND-2000, Thermofisher) according to the manufacturer instructions. The DOL was of 3 and 3.4 AF per mole of N or aggregated IFX, respectively.

### 4.12 Internalization assay

Immature moDCs were incubated (2 x10^5^ cells/200μL) with each labelled IFX N or UV (200µg/ml) in RPMI-1640 medium at 4°C (for binding) or 37°C (for internalization) for 2 hours. Cells were then washed to remove excess free N or aggregated IFX then introduced into an Attune Nxt cytometer (Invitrogen) to assess internalization. For inhibition experiments, moDCs were pre-treated for 1 hour with the respective blocking agents cytochalasin D (C2618, Sigma), chlorpromazine (C8138, Sigma), amiloride (A7410, Sigma), mannan (M7504, Sigma), FcγRIIa (clone IV.3, 60012, STEMCELL Technologies), anti-CD206 (321102, Biolegend), before addition of labelled N or aggregated IFX for 120 min. The internalization rate was calculated from the difference (delta) between the mean fluorescent intensity (MFI) of surface-localized (4°C) and internalized (37°C) IFX N or UV and evaluated by the percentage of internalizing cells. For inhibition experiments, results were expressed as relative uptake and relative percentage of internalizing cells calculated by using the following formulas:


$$\text{Relative uptake}=\frac{\text{delta MFI inhibition condition }}{\text{delta MFI without inhibitor}}$$

$$\text{Relative percentage}=\frac{\text{AF positive cells inhibition condition }}{\text{AF positive cells without inhibitor}}$$

### 4.13 Confocal laser microscopy and imaging analysis

Confocal microscopy was used to determine the routing of IFX N or UV intro intracellular compartments. For this, moDCs were incubated with AF-labelled IFX N or UV IFX (200µg/ml), then washed, fixed and permeabilized (ThermoFisher Scientific). After a blocking step with 2% BSA in PBS, moDCs were incubated with marker antibodies for early endosome antigen 1 (EEA-1) (C45B10, Cell Signaling), lysosomal associated membrane protein -1 (LAMP-1) (C54H11, Cell signaling) or HLA-DR (307602, Biolegend) followed by either a goat anti-Rabbit IgG (H+L) Cross-Adsorbed Secondary Antibody, Alexa Fluor™ 647 (A-21244, Thermo Fisher Scientific) or a rabbit anti-M IgG (H+L) Cross-Adsorbed Secondary Antibody, Alexa Fluor™ 647 (A-21239,Thermo Fisher Scientific). Stained moDCs were mounted in Fluoromount-G™ Mounting Medium (00-4958-02, Thermo Fisher Scientific) then poured to the bottom of an IBIDI µ-Slide 8 Well high (80801, Ibidi). Samples were imaged with an inverted TCS SP8 gated-STED confocal microscope (Leica, Germany) operating with LAS X v3.5.5 software and using a HC PL APO CS2 63 ×/1.40 oil immersion objective. For fluorophore excitation, the instrument was equipped with a WLL laser set at 490 nm for AF488 (green channel) and 647 nm for AF647 (red channel).

Green and red channels fluorescence emissions were collected using Internal Hybrid Detectors (HyD) using gating in a sequential mode with variable beam splitter set respectively between 500–574 and 654–800 nm. PMT detectors were used for Brightfield channel and for others. The pinhole was set at 1.0 Airy unit giving an optical thickness of 0.89 µm for each slice. Stacks were collected with 0.30 μm intervals along the z axis.

The line tool of ImageJ software (version 1.42, NIH) (81) was used to delineate the area of the analyzed cell. To quantify the degree of colocalization between fluorescently labeled IFX N or UV and EEA-1, LAMP-1 or HLA-DR, the Pearson’s correlation coefficient (PCC) and the Manders’ correlation coefficient (M1 and M2) were determined from the corresponding confocal images using FIJI software (ImageJ, National Institutes of Health, Bethesda, MD, USA) with EZ Colocalization plugin. We used an approach without Costes randomizations, taking the automatic adjusted threshold for the two different channels. The cell contours were manually defined based on the EEA-1, LAMP-1 or HLA-DR channel to select the region of interest (ROI) for the analysis and avoid background signals. In the script run for each channel the automatic threshold was adjusted, where channel 1 represented EEA-1, LAMP-1 or HLA-DR and channel 2 corresponded to IFX N or UV. All the images were analyzed with the same script and the threshold of each individual channel was adjusted each time. From the EZ Colocalization analysis, we extracted Pearson’s and Manders’ (M1 and M2) correlation coefficients for data representation. Quantitative values were exported to Prism for further analysis and graphical representation.

### 4.14 Immunoblot analysis

moDCs were incubated for 5 min with IFX N or UV at 200µg/ml, harvested and washed in cold PBS before lysis in 30 ml of lysis buffer (20 mM Tris [pH 7.4], 137 mM NaCl, 2 mM EDTA [pH 7.4], 2 mM sodium pyrophosphate, 1% Triton, 10% glycerol, 1 mM PMSF, 1 mM Na3VO4, 25 mM b glycerophosphate, 10 mg/ml aprotinin, 10 mg/ml leupeptin, and 100 mg/ml pepstatin). After centrifugation at 15,000 rpm for 20 min at 4 °C, 30µg of denatured protein were loaded onto 10% SDS-PAGE gels and transferred to nitrocellulose membranes, which were successively incubated with Abs directed against EEA-1(C45B10, Cell Signaling). Membranes were then incubated with an HRP-coupled Ab against ß-actin as a loading control (12262S, Cell signaling). Immunoreactive bands were detected by their chemiluminescence using the ChemiDoc XRS+ System (Bio-Rad Laboratories). Bands were quantified with Image Lab software and normalized with the loading control.

### 4.15 Cytokines and chemokines quantification in cell culture supernatants

moDCs were stimulated for 24 hours with IFX N or UV (200µg/ml). Meso Scale Discovery (Meso Scale Diagnostics) multiplex assay was performed on culture supernatants, according to the manufacturer’s instructions, to measure in duplicate IL-6, IL-8, IL-10, IL-12/p40, CCL3 (MIP-1a) and CCL4 (MIP-1b). The quantification ranges were as follows: IL-6, 0.49–2040 pg/ml; IL-8, 0.56–2300 pg/ml; IL-10, 0.93–3820 pg/ml; IL-12/23p40, 4.85–19,900 pg/ml; CCL3, 1.44–5920 pg/ml and CCL4, 0.53–2200 pg/ml.

### 4.16 Ex vivo T cell proliferation assay

CD4+ T cells were isolated from PBMCs by positive selection with MidiMACS separation columns and anti-CD4 Abs coated on magnetic beads (130-045-101, Miltenyi Biotec). These T cells were confirmed to have purity of 95%, based on CD4 expression evaluated by flow cytometry (561841, BD Biosciences). CD4+ T lymphocytes were labeled with 0.5 mM CFSE (C34570, Thermo Fischer Scientific), following the manufacturer’s instructions. moDCs were pre-treated or not with mannan (2mg/ml) then stimulated with IFX N or IFX UV for 16 hours, then washed and cocultured with autologous CD4+ T cells at a 1:5 DC/T cell ratio for 5 days, in RPMI 1640 GlutaMAX supplemented with 5% human serum in round-bottom 96-well plates. CFSE fluorescence was analyzed using flow cytometry with an Attune Nxt (Invitrogen) using FlowJo software (version 10).

### 4.17 Statistical analysis

Statistical analyses were performed using GraphPad Prism software. Comparisons between two paired groups were performed using either a paired Student’s t-test or a Wilcoxon matched-pairs signed-rank test, as appropriate. Comparisons among multiple groups were performed using the Kruskal–Wallis test followed by Dunn’s multiple comparison test followed by Dunn’s multiple comparison test, as appropriate. A p value<0.05 was considered statistically significant.

## Data Availability

The raw data supporting the conclusions of this article will be made available by the authors, without undue reservation.
