# Supplementary material for: Mechanisms of therapeutic antibodies immunogenicity: infliximab oligomeric aggregates increase T-cell response and uptake by dendritic cells involving mannose-sensitive receptor CD206
Source: Front Immunol. 2026 Sep 3;17:1904926. doi: 10.3389/fimmu.2026.1904926 (PMC13582642; doi:10.3389/fimmu.2026.1904926)
Supplement: Supplementary file 1 [file DataSheet1.pdf]

(a)

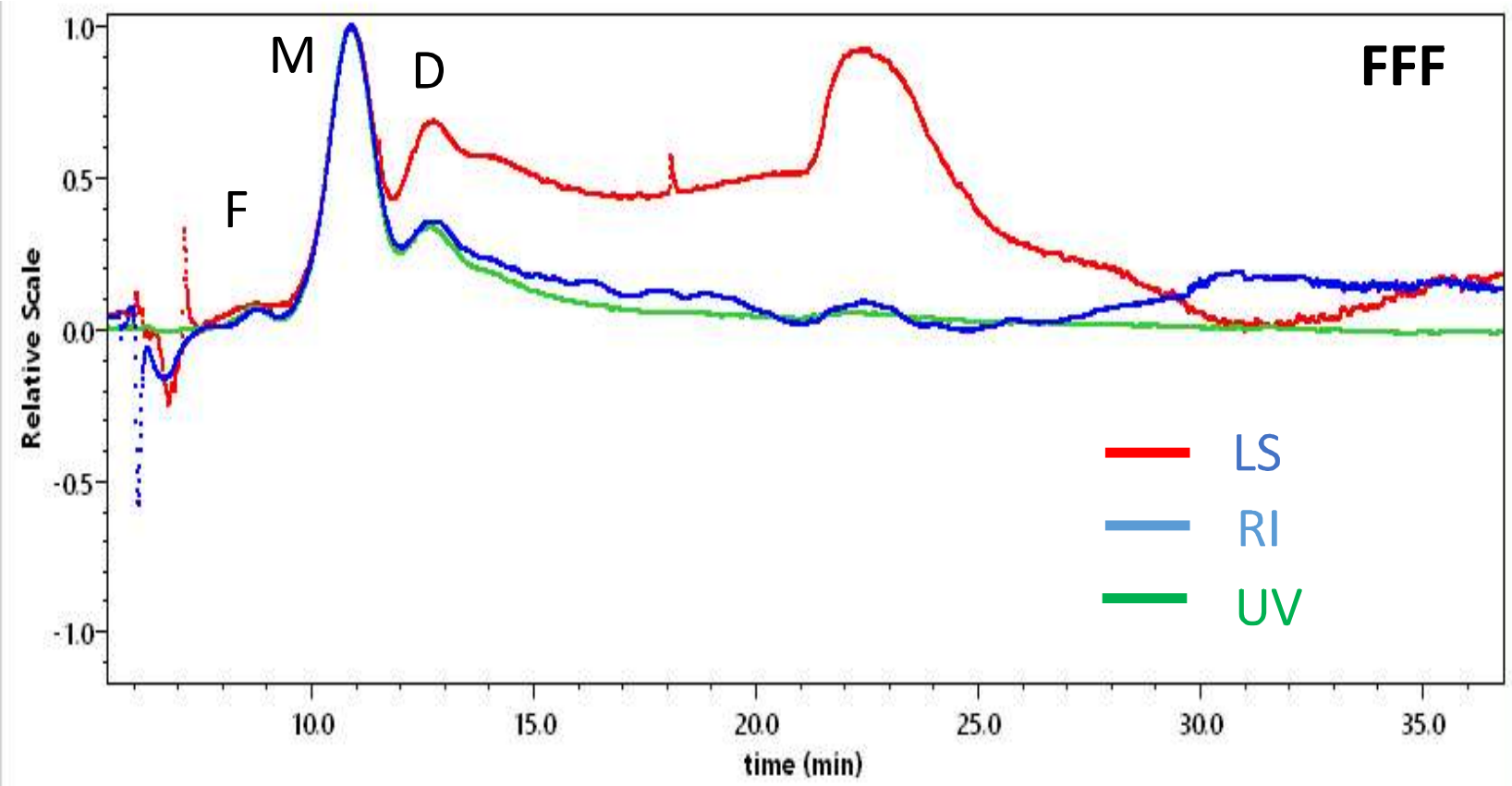

(b)

	Percentages
**Monomer**	**52.8%**
**Dimers**	**22.4%**
**Trimers/ Oligomers**	**24.7%**
**Fragment**	**2.1%**

(c)

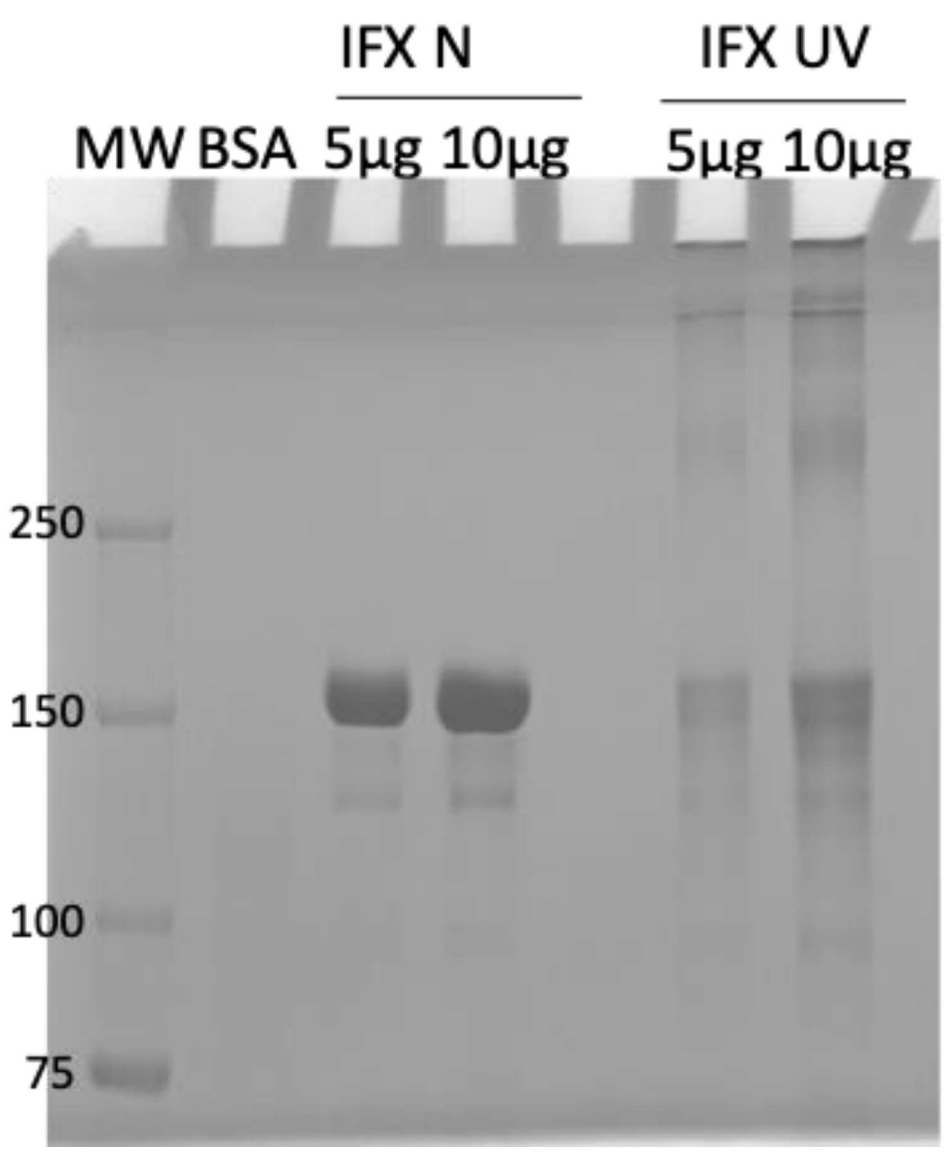

(d)

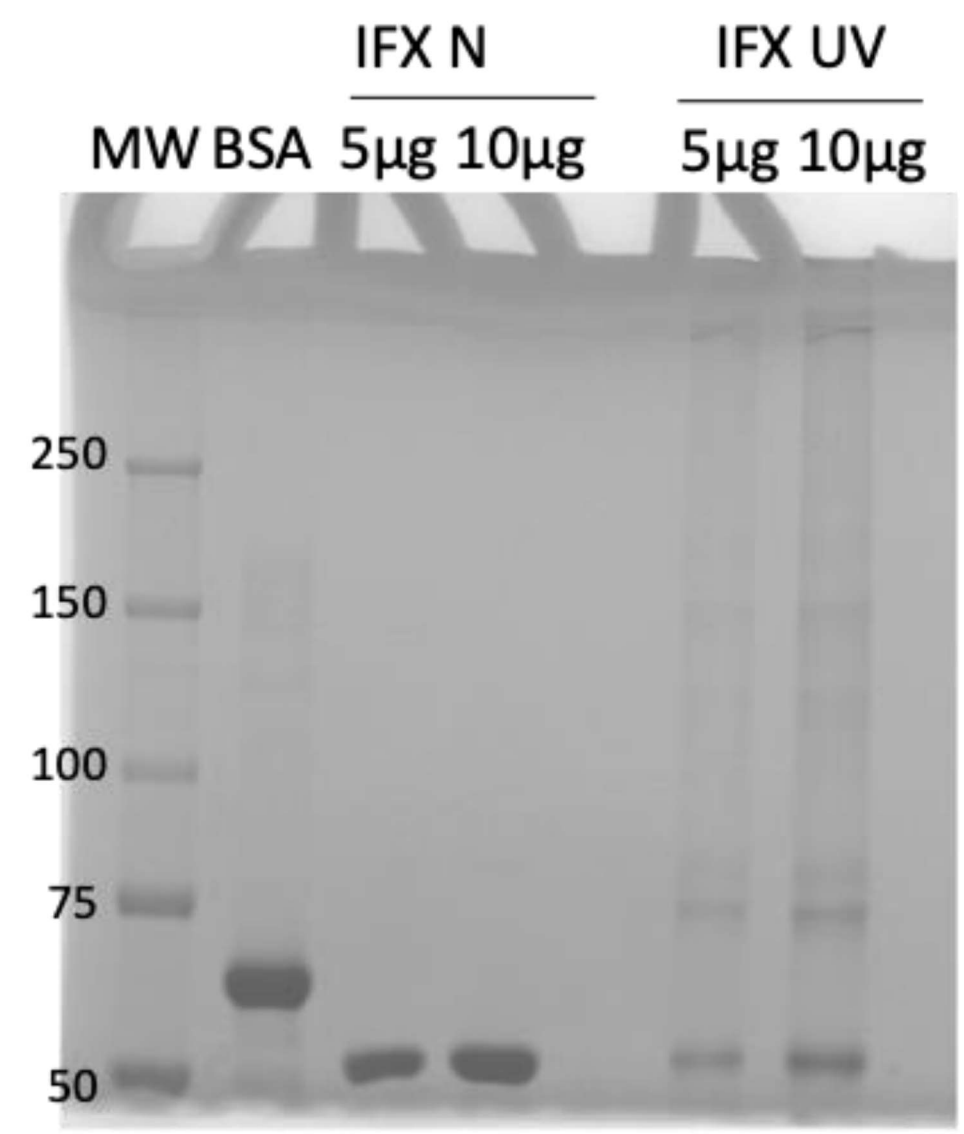

Supplementary data 1: Characterization of infliximab aggregates

(a-b) Characterization by Field Flow Fractionation (FFF)- multi-angle light scattering (MALS) of native (N) or aggregated (UV) infliximab (IFX). (a) Typical chromatograms showing the ultraviolet (UV), refractive index (RI) and the MALS signals; (n=2; two independent experiments); (b) percentages of different species found following UV treatment and calculated after FFF-MALS analysis

(c-d) SDS-PAGE of IFX N and IFX UV. Protein samples in SDS buffer were subjected to electrophoresis under non-reducing (c) and reducing conditions (d) The gel was stained with Coomassie blue G-250. Molecular mass markers (MW) are indicated in kilodaltons. Bovine serum albumin (BSA) was taken as a mass molecular weight control, in addition to the MW; (representative gel, several aggregated preparations were tested over time)
