# Supplementary material for: Mechanisms of therapeutic antibodies immunogenicity: infliximab oligomeric aggregates increase T-cell response and uptake by dendritic cells involving mannose-sensitive receptor CD206
Source: Front Immunol. 2026 Sep 3;17:1904926. doi: 10.3389/fimmu.2026.1904926 (PMC13582642; doi:10.3389/fimmu.2026.1904926)
Supplement: Supplementary file 2 [file DataSheet2.pdf]

(a)

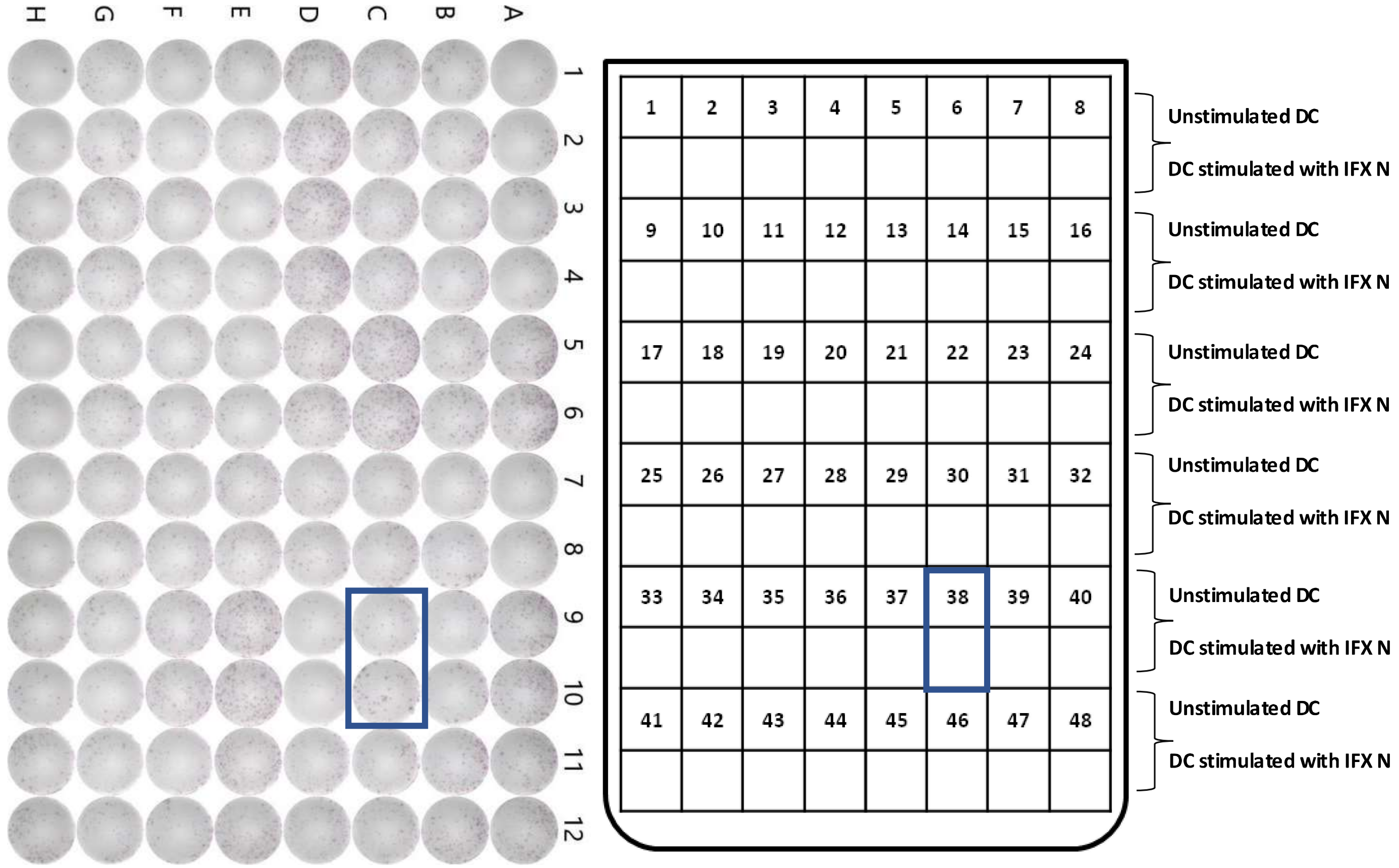


(b)

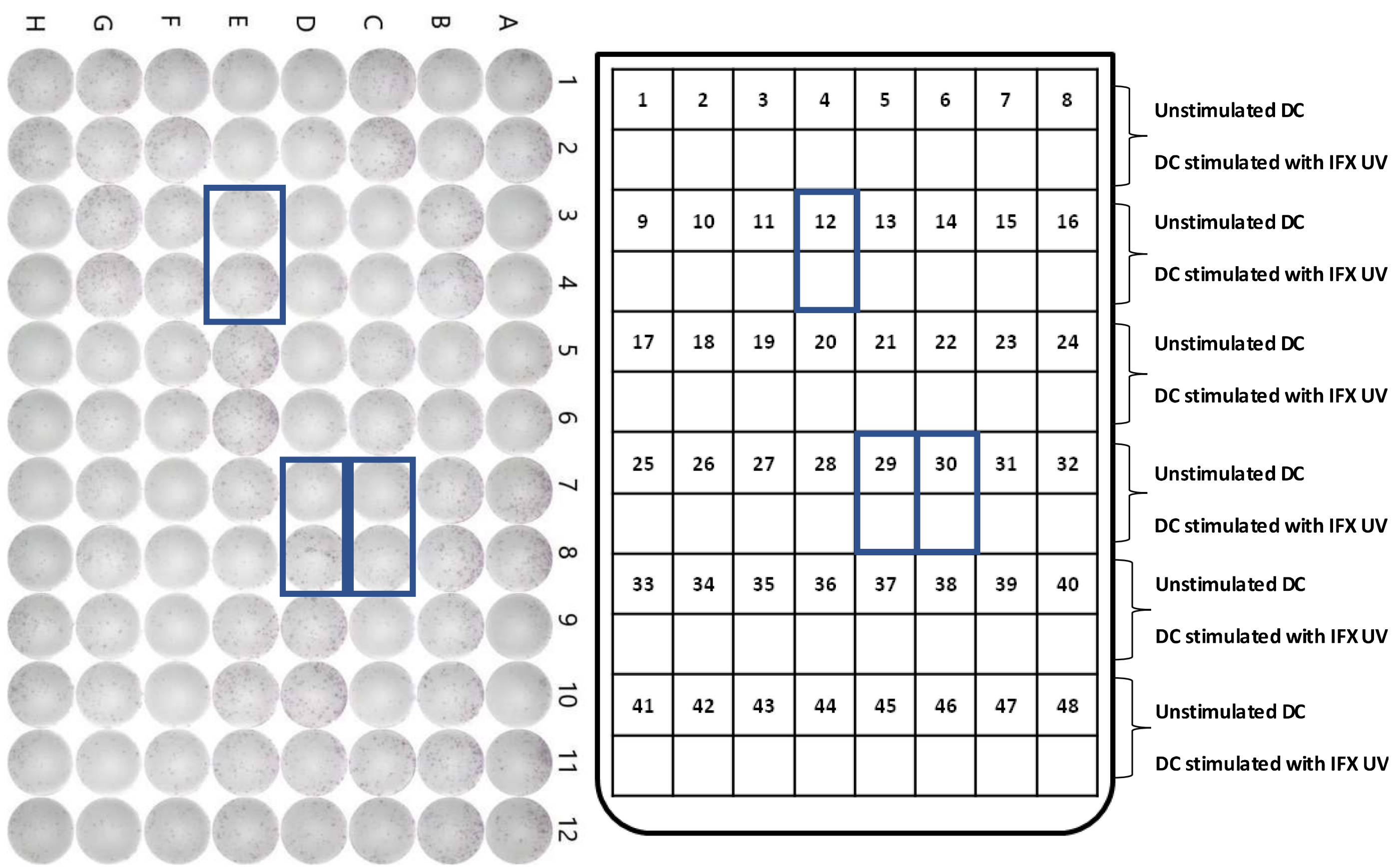


**Supplementary Data 2. Full ELISpot plate corresponding to figure 2a**.

*IFN-γ ELISpot response of naïve CD4⁺ T cells from donor PL11 stimulated with unloaded DC or DC loaded with IFX N (a) or IFX UV (b). The image shows the complete, unedited plate from which the positive wells presented in figure 2a were selected.*
