# Supplementary material for: Mechanisms of therapeutic antibodies immunogenicity: infliximab oligomeric aggregates increase T-cell response and uptake by dendritic cells involving mannose-sensitive receptor CD206
Source: Front Immunol. 2026 Sep 3;17:1904926. doi: 10.3389/fimmu.2026.1904926 (PMC13582642; doi:10.3389/fimmu.2026.1904926)
Supplement: Supplementary file 3 [file DataSheet3.pdf]

(a)

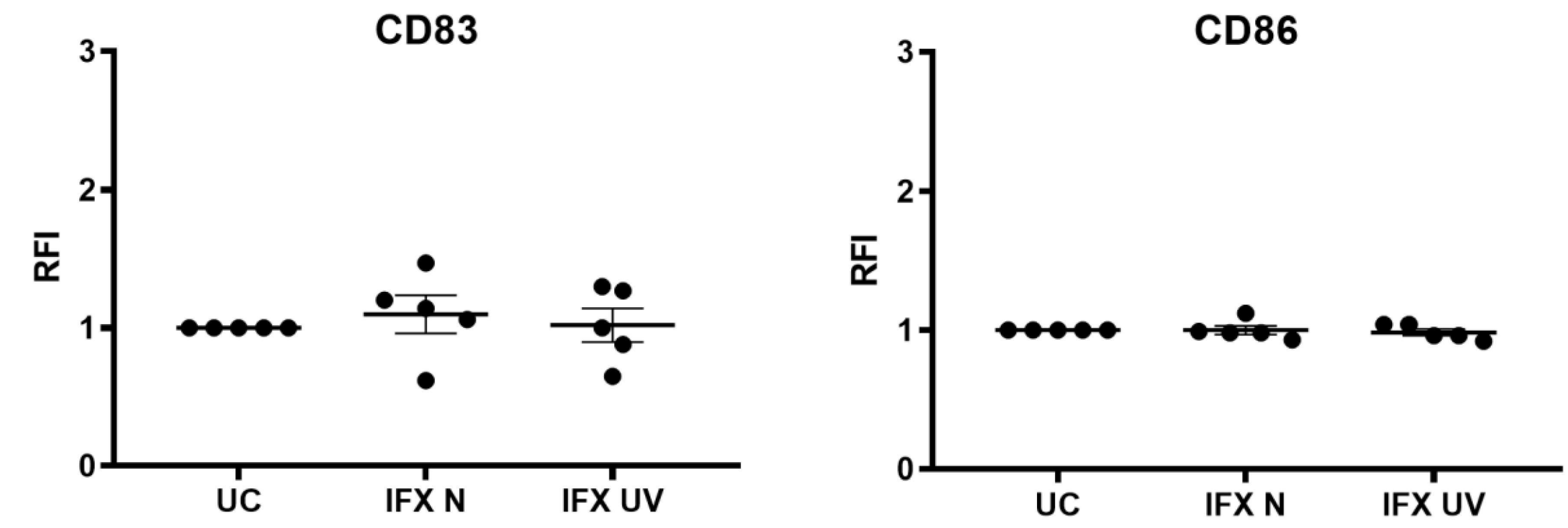

(b)

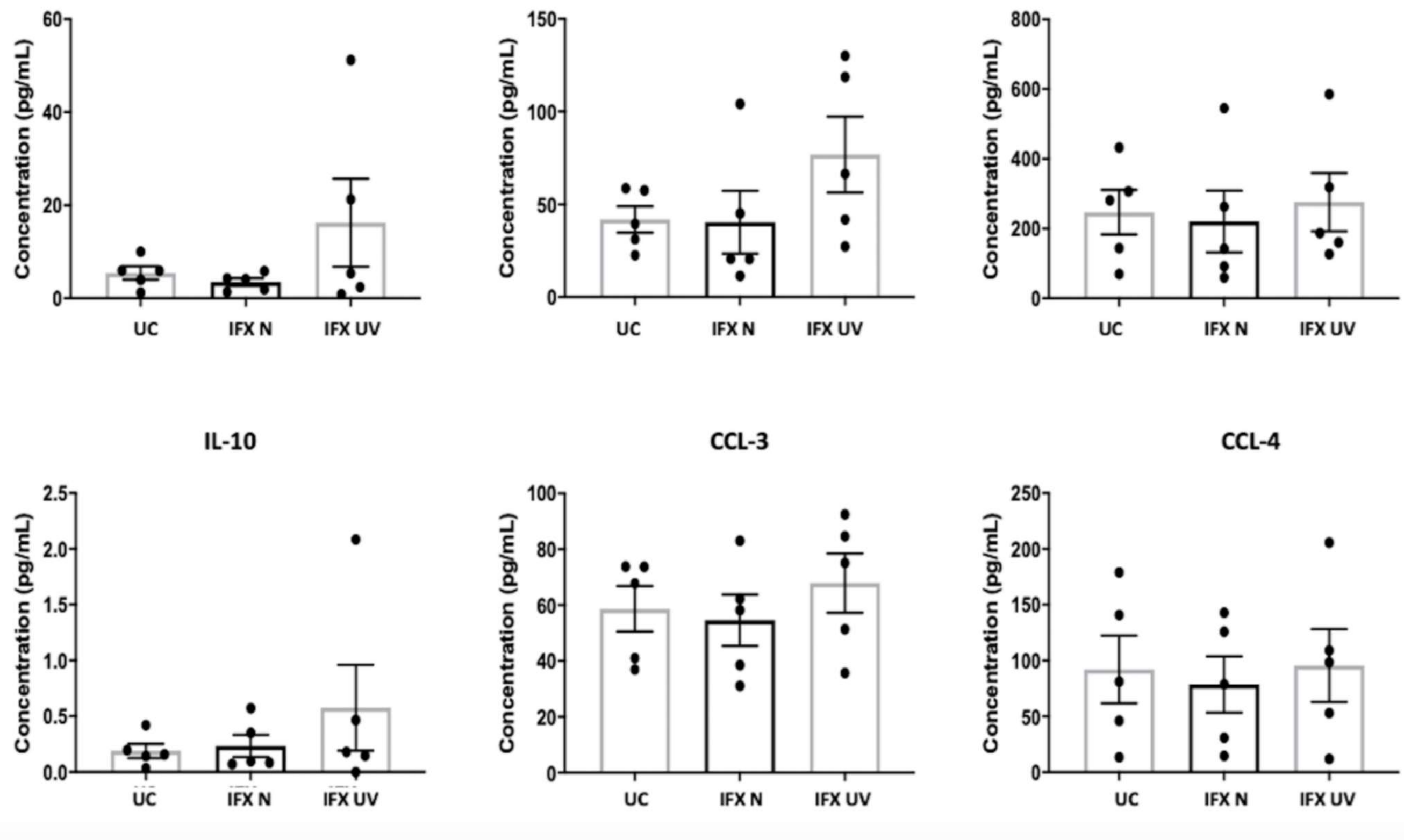

Supplementary data 3: IFX UV does not modifiy moDCs phenotype nor cytokines and chemokines production.

(a) Immature moDCs were treated for 24 hours with IFX N or IFX UV (200 µg/ml). Cells were then collected, washed, and analyzed by flow cytometry for surface marker expression. Results of 6 independent experiments are expressed as RFI compared with untreated control (UC) and represented as dot plot.

(b) Immature moDC were treated for 24 hours with IFN N or UV. Cytokine and chemokine concentrations in culture supernatants were measured in duplicate using an MSD multiplex assay. Detection limits are indicated in Materials and Methods. Results of 5 independent experiments are represented as dot plot.
