# Supplementary material for: Mechanisms of therapeutic antibodies immunogenicity: infliximab oligomeric aggregates increase T-cell response and uptake by dendritic cells involving mannose-sensitive receptor CD206
Source: Front Immunol. 2026 Sep 3;17:1904926. doi: 10.3389/fimmu.2026.1904926 (PMC13582642; doi:10.3389/fimmu.2026.1904926)
Supplement: Supplementary file 4 [file DataSheet4.pdf]

(a)

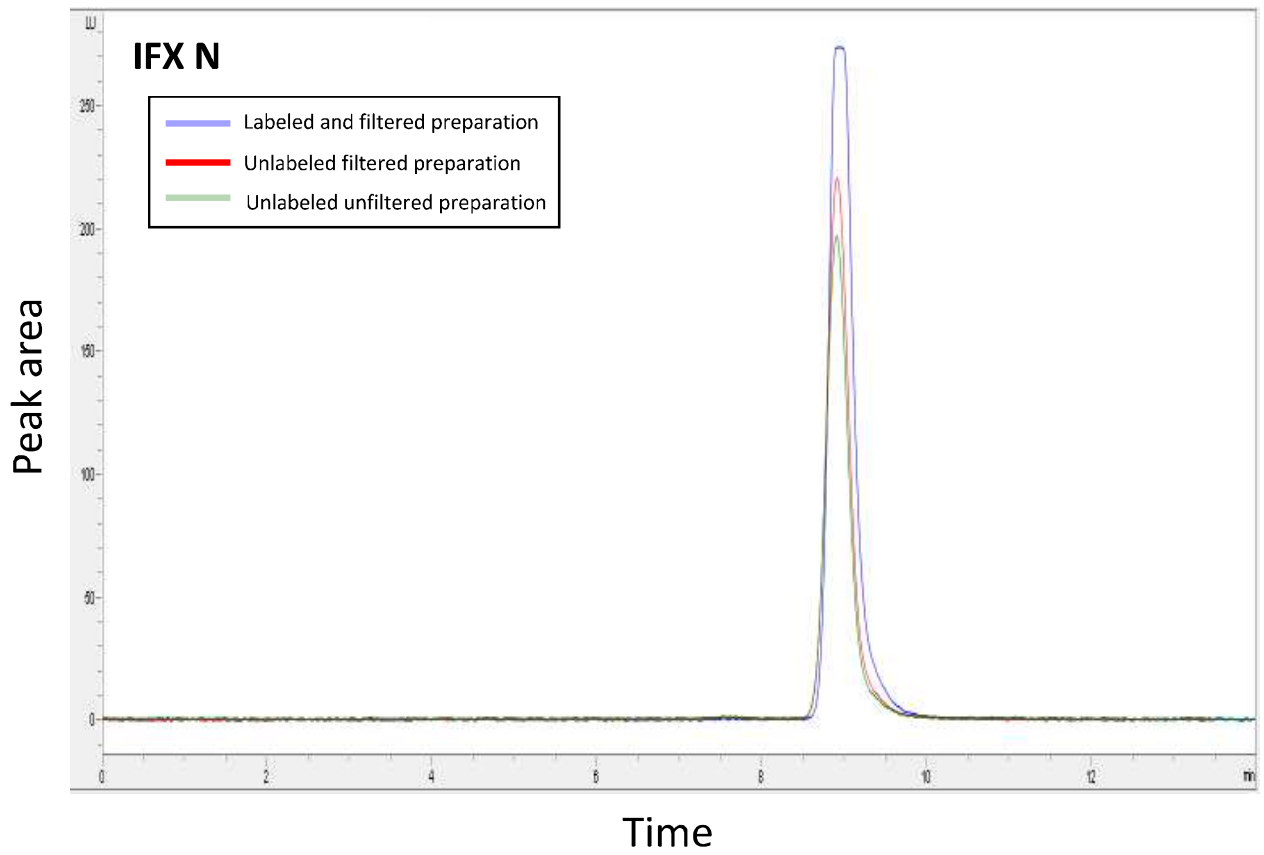

(b)

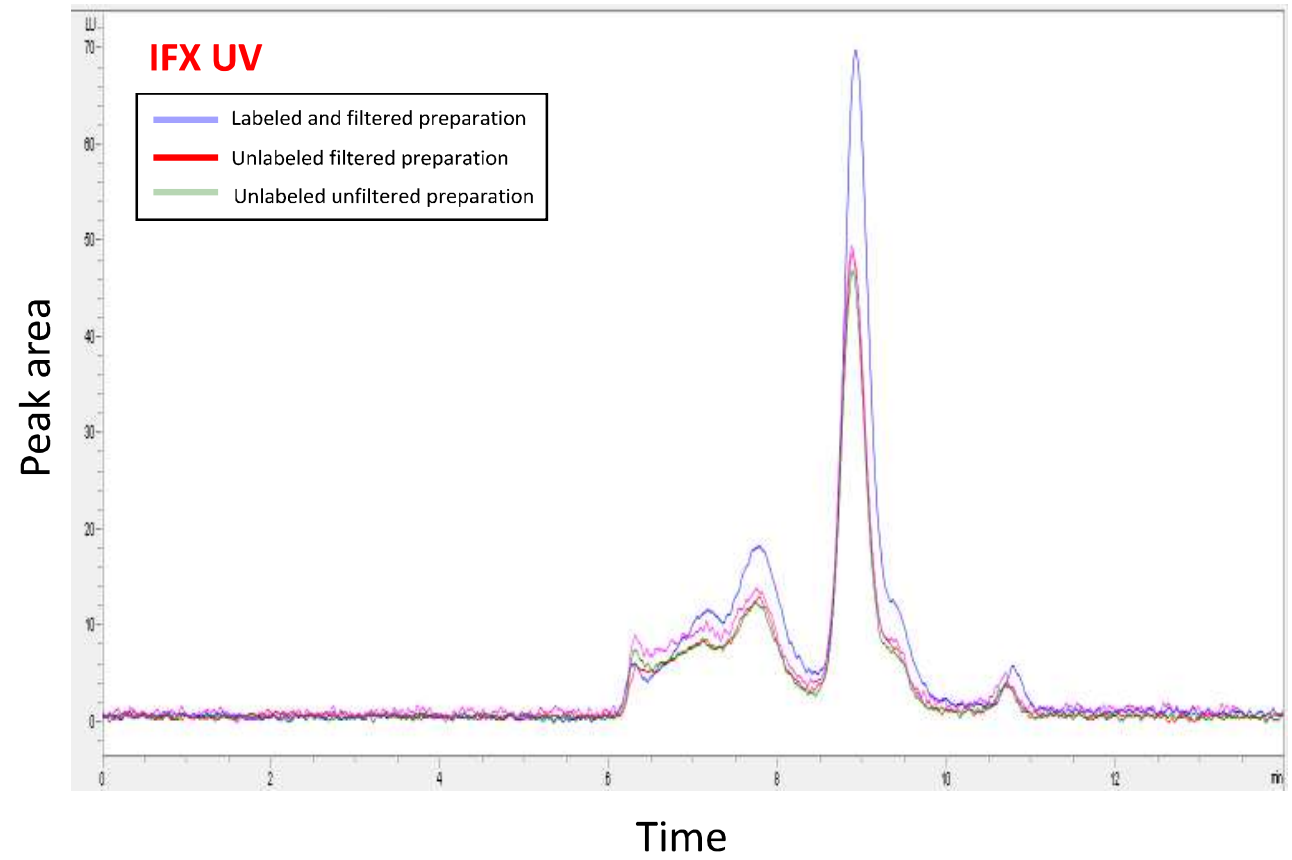

(c)

Receptor	Percentages
Monomer	54.4% ±0.2%
Dimers	22.9% ±0.1%
Trimers	22.7% ±0.4%

Supplementary data 4: Characterization of Alexa-Fluor 488 labelled native and aggregated IFX

Chromatograms of labelled (green line), filtered (red line) or native (N) (a) or aggregated (UV) (b) infliximab (IFX) (blue line) (1mg/ml) were characterized by size exclusion chromatography. (c) percentages of different species calculated after SEC analysis (n=2)
