# Supplementary material for: Mechanisms of therapeutic antibodies immunogenicity: infliximab oligomeric aggregates increase T-cell response and uptake by dendritic cells involving mannose-sensitive receptor CD206
Source: Front Immunol. 2026 Sep 3;17:1904926. doi: 10.3389/fimmu.2026.1904926 (PMC13582642; doi:10.3389/fimmu.2026.1904926)
Supplement: Supplementary file 5 [file DataSheet5.pdf]

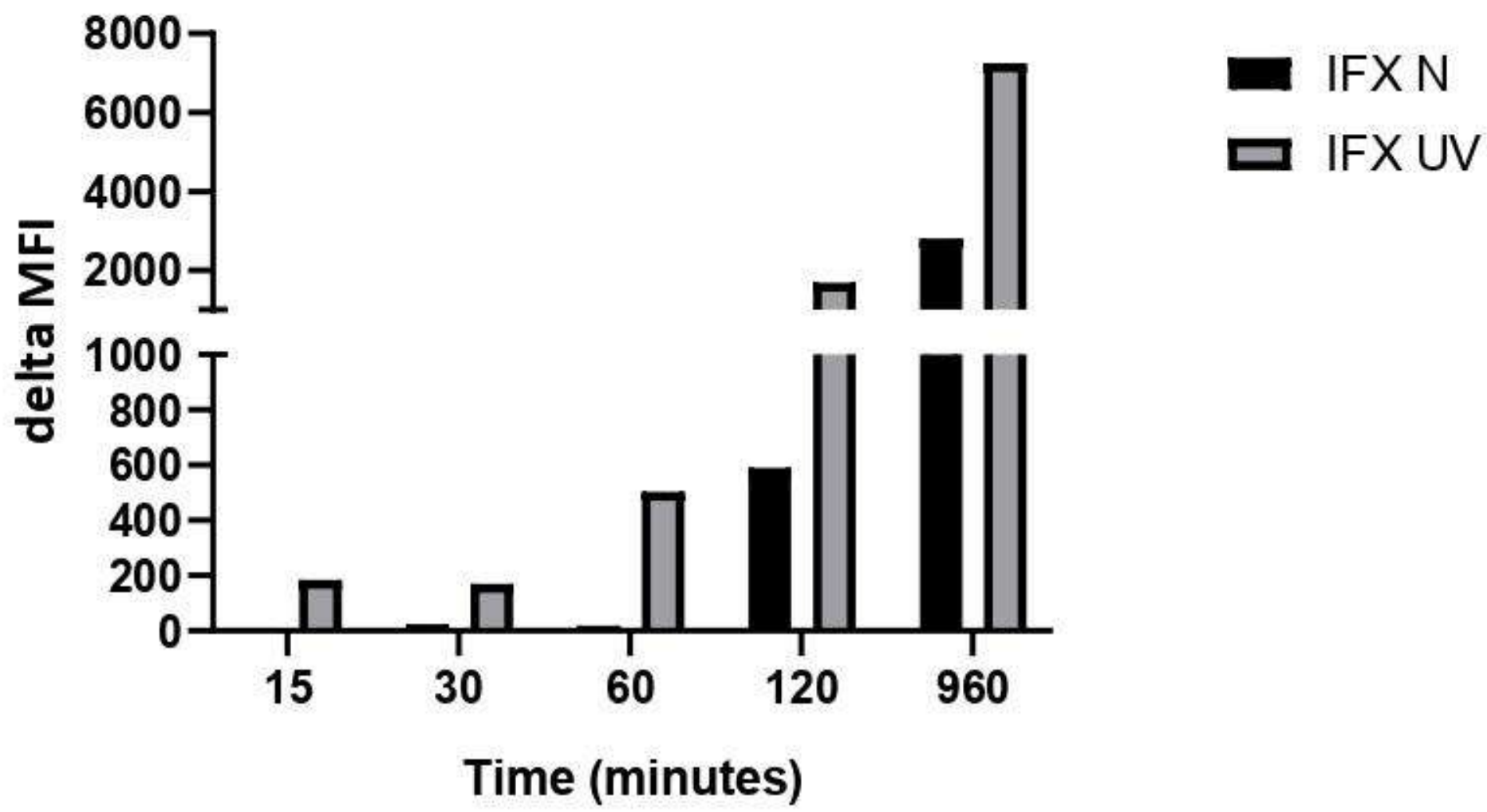

Supplementary data 5: Kinetic of IFX N and UV internalization

moDC were incubated for 5, 30, 60, 120 or 960 minutes with Alexa Fluor 488 (AF-488) labelled native (N) or aggregated (UV) infliximab (IFX) at 37°C or 4°C ;

Internalization rate was evaluated by flow cytometry and expressed as delta of the mean fluorescent intensity (MFI), calculated from the difference between the MFI of surface-localized (4°C) (n=2)
