# Supplementary material for: Mechanisms of therapeutic antibodies immunogenicity: infliximab oligomeric aggregates increase T-cell response and uptake by dendritic cells involving mannose-sensitive receptor CD206
Source: Front Immunol. 2026 Sep 3;17:1904926. doi: 10.3389/fimmu.2026.1904926 (PMC13582642; doi:10.3389/fimmu.2026.1904926)
Supplement: Supplementary file 6 [file DataSheet6.pdf]

(a)

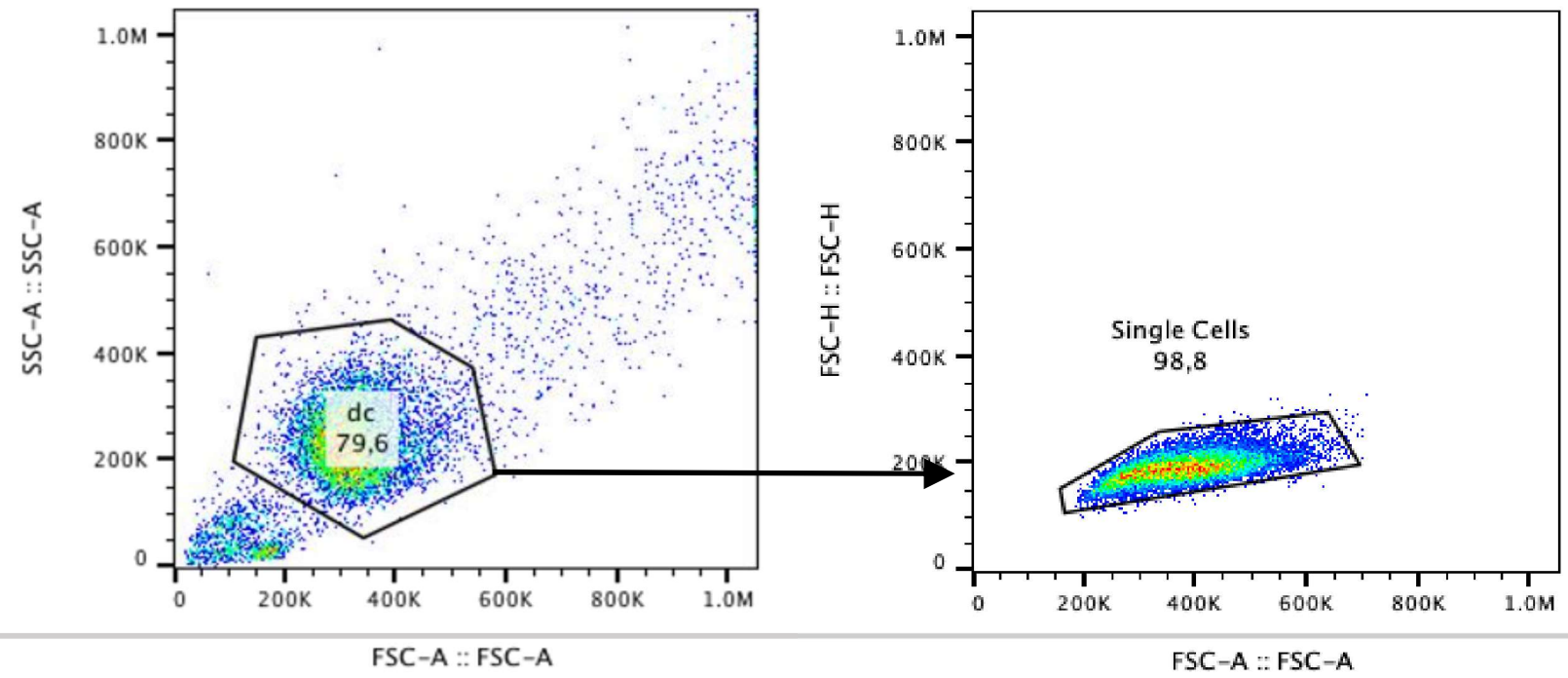

(b)

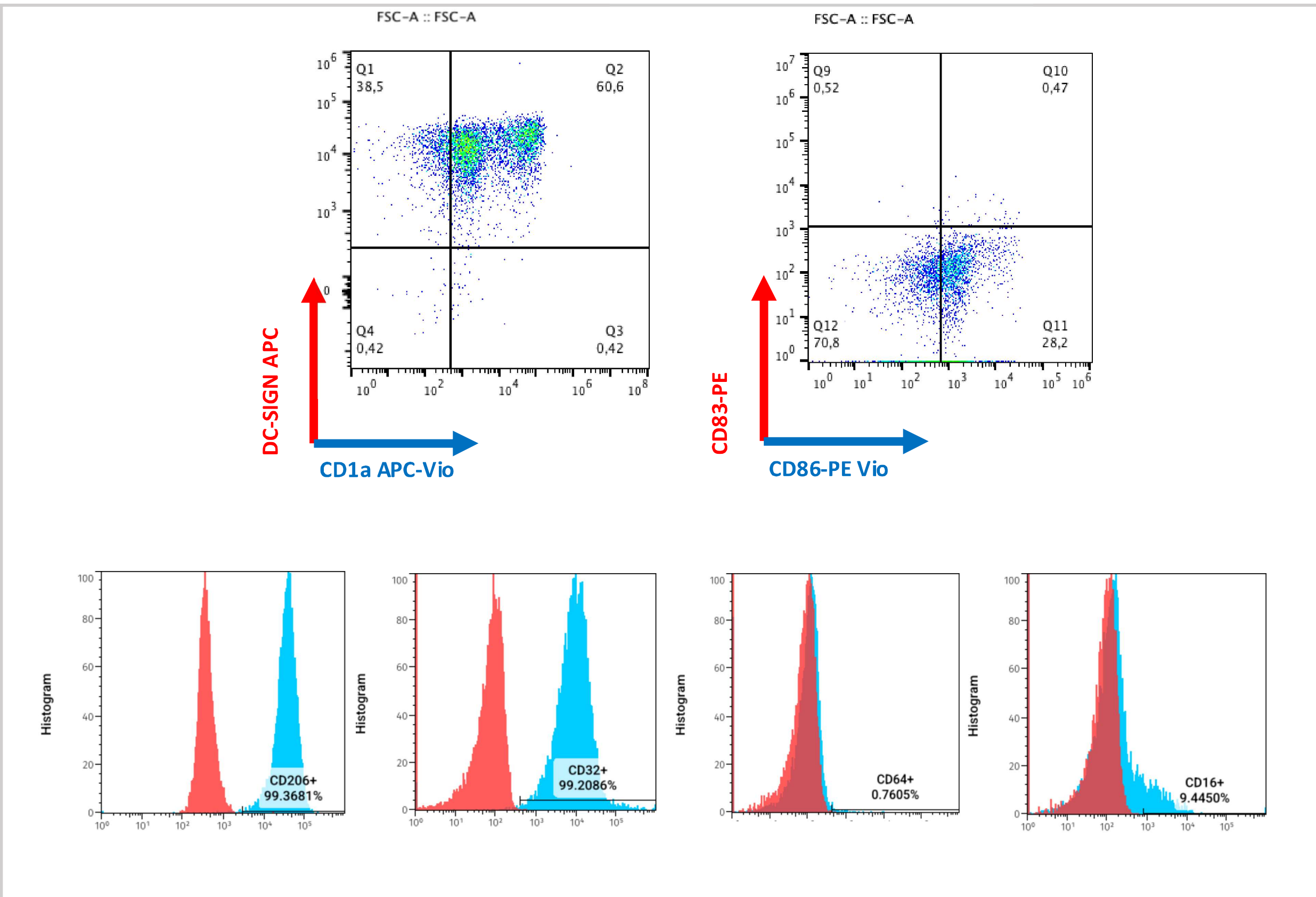

(c)

Receptor	Expression
DC-SIGN	95% ±5%
CD206	90% ±5%
CD64	<10%
CD32	95% ±5%
CD16	<10%

Supplementary data 6: Receptors expressed by moDCs

Immature moDCs were collected after differentiation, washed, and analyzed by flow cytometry for surface marker expression.

(a) Gating strategy; (b) Representative result; (c) Percentage of expression of different internalization receptors by our moDCs model (n=6)
