# Supplementary material for: Mechanisms of therapeutic antibodies immunogenicity: infliximab oligomeric aggregates increase T-cell response and uptake by dendritic cells involving mannose-sensitive receptor CD206
Source: Front Immunol. 2026 Sep 3;17:1904926. doi: 10.3389/fimmu.2026.1904926 (PMC13582642; doi:10.3389/fimmu.2026.1904926)
Supplement: Supplementary file 7 [file DataSheet7.pdf]

(a)

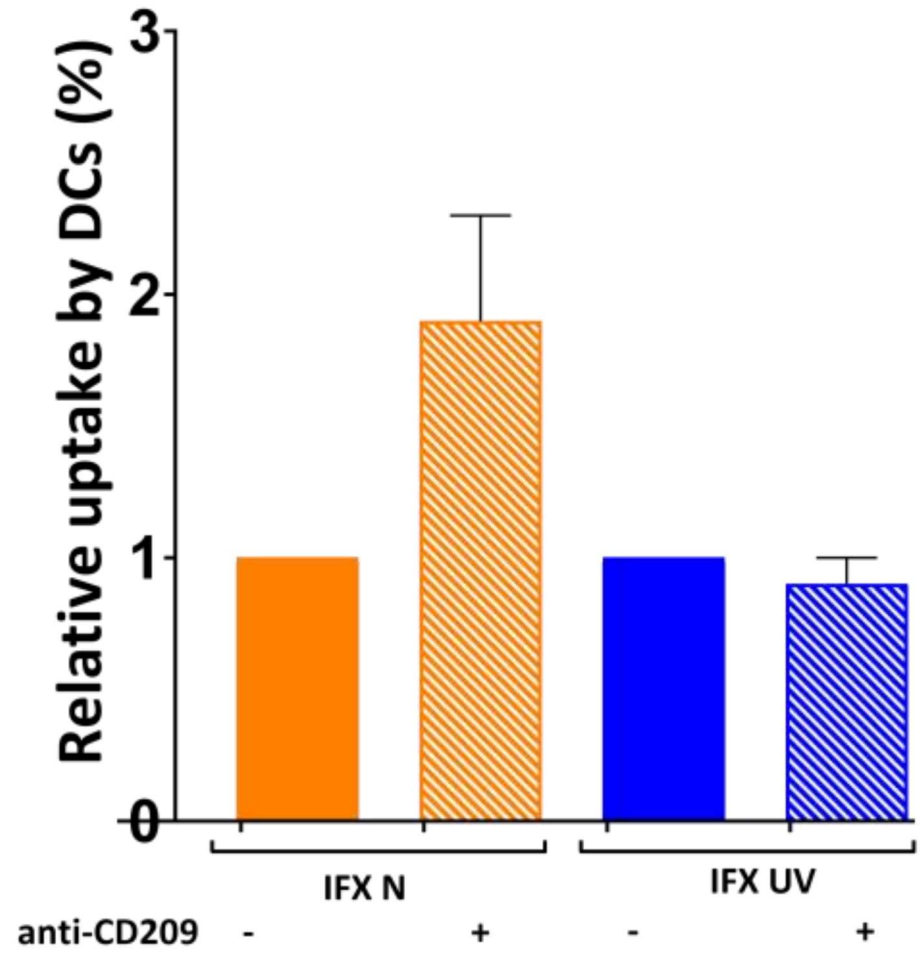

(b)

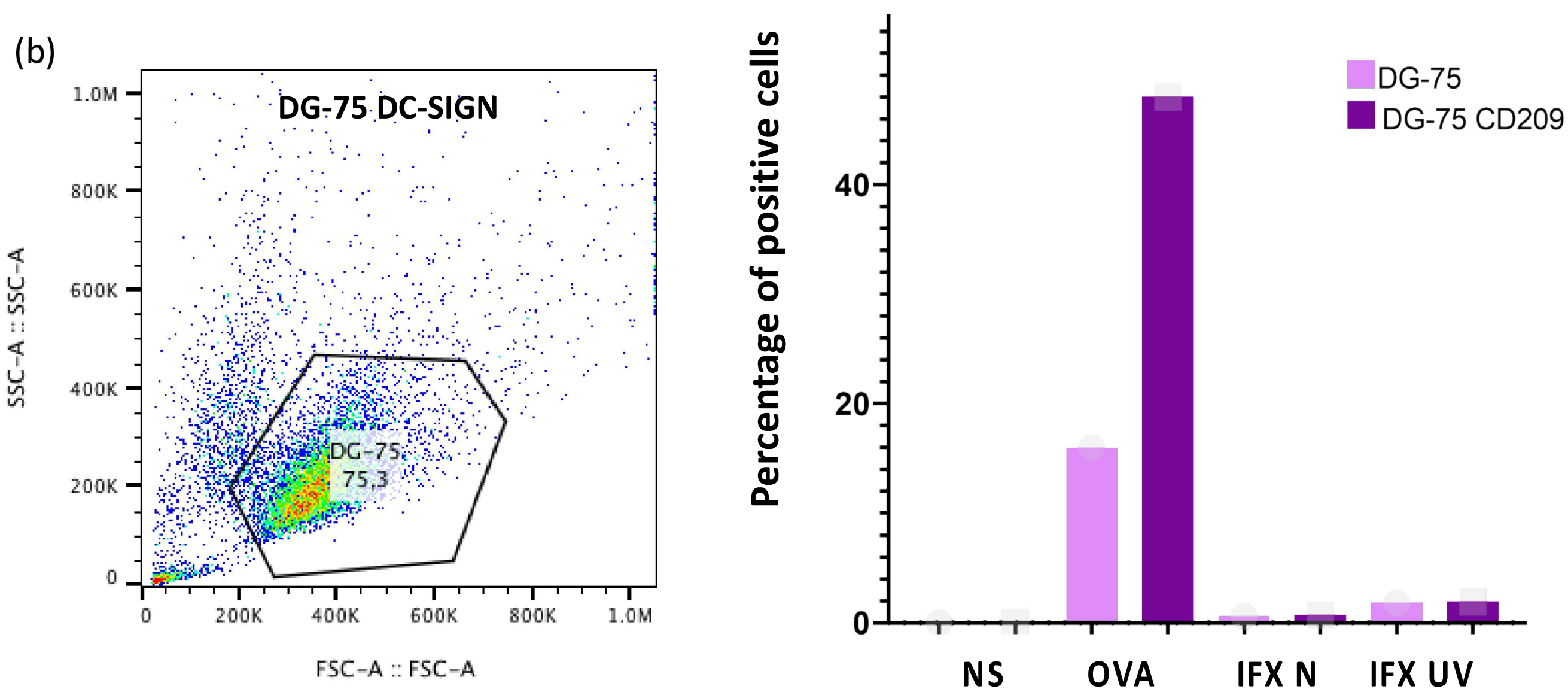

Supplementary data 7: IFX N or UV internalization is not CD209-mediated

(a)moDCs were pre-treated for 1 hour with anti-CD209 before incubation with fluorescently labelled IFX N or IFX UV. Results are expressed as relative uptake of IFX N or IFX UV by DCs defined as (37°C MFIinh-4°C MFIinh)/ (37°C MFIcondition -4°C MFIcondition), where "MFIinh" stands for MFI detected in the presence of the inhibitors and "MFI condition" stands for MFI detected in the presence of IFX N or UV (n=2);

(b) DG-75 DC-SIGN- or DG-75 DC-SIGN+ were incubated for 2 hours with Alexa Fluor 488 (AF-488) labelled ovalbumine (OVA) native (N) or aggregated (UV) infliximab (IFX) at 37°C or 4°C; Results are expressed as the percentage of positive cells, as MFI values were very low; however, these data are shown to illustrate the proportion of cells displaying detectable fluorescence.
