# Supplementary material for: Mechanisms of therapeutic antibodies immunogenicity: infliximab oligomeric aggregates increase T-cell response and uptake by dendritic cells involving mannose-sensitive receptor CD206
Source: Front Immunol. 2026 Sep 3;17:1904926. doi: 10.3389/fimmu.2026.1904926 (PMC13582642; doi:10.3389/fimmu.2026.1904926)
Supplement: Supplementary file 8 [file DataSheet8.pdf]

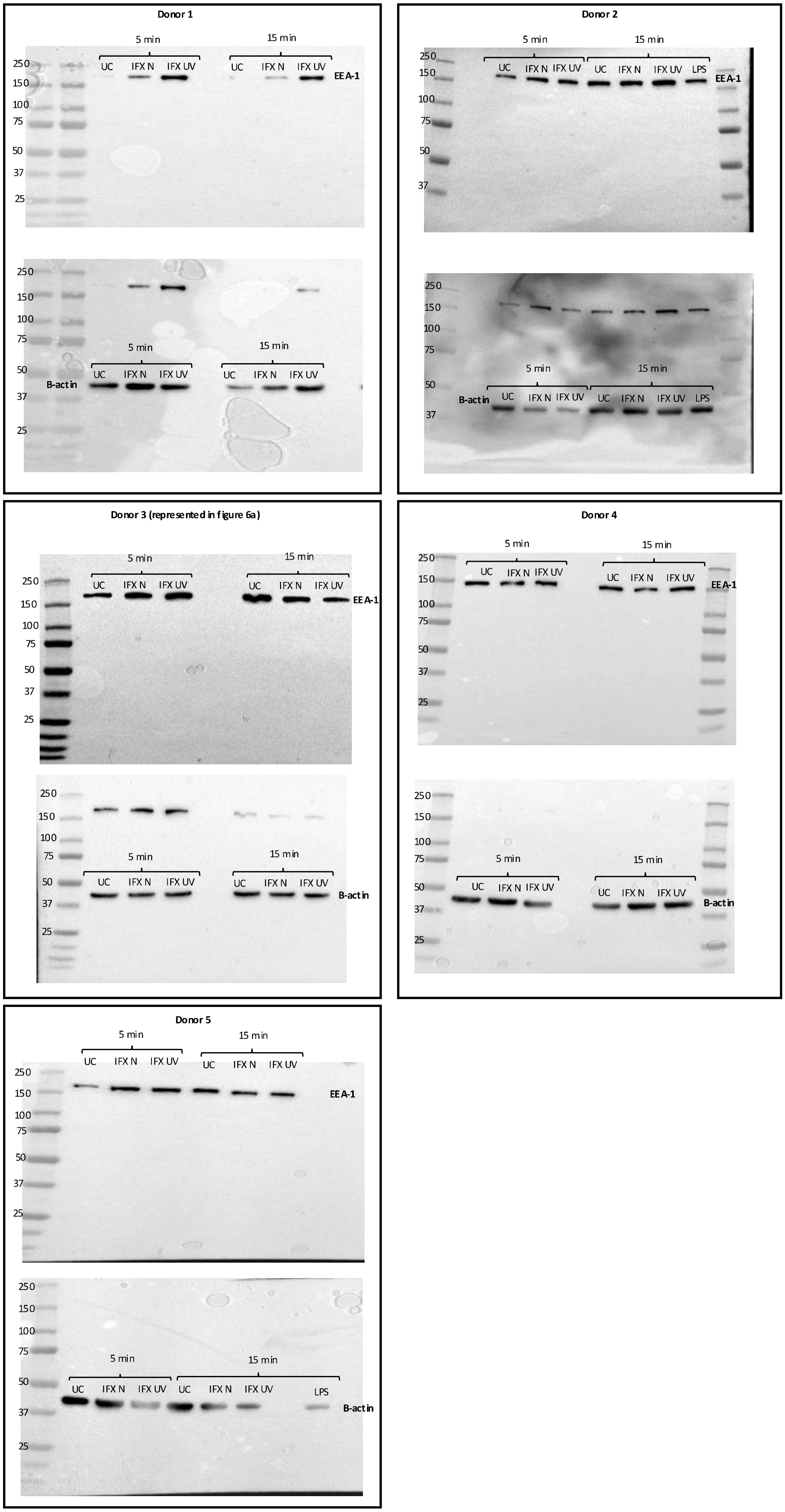

Supplementary data 8: Original western blots from figure 6a.

The original, uncropped western blot membrane scans are shown. Molecular weight markers are included. Full scans show EEA-1 expression in untreated moDCs or moDCs treated with native (IFX N) or aggregated (IFX UV) infliximab.
