# Supplementary material for: Mechanisms of therapeutic antibodies immunogenicity: infliximab oligomeric aggregates increase T-cell response and uptake by dendritic cells involving mannose-sensitive receptor CD206
Source: Front Immunol. 2026 Sep 3;17:1904926. doi: 10.3389/fimmu.2026.1904926 (PMC13582642; doi:10.3389/fimmu.2026.1904926)
Supplement: Supplementary file 9 [file DataSheet9.pdf]

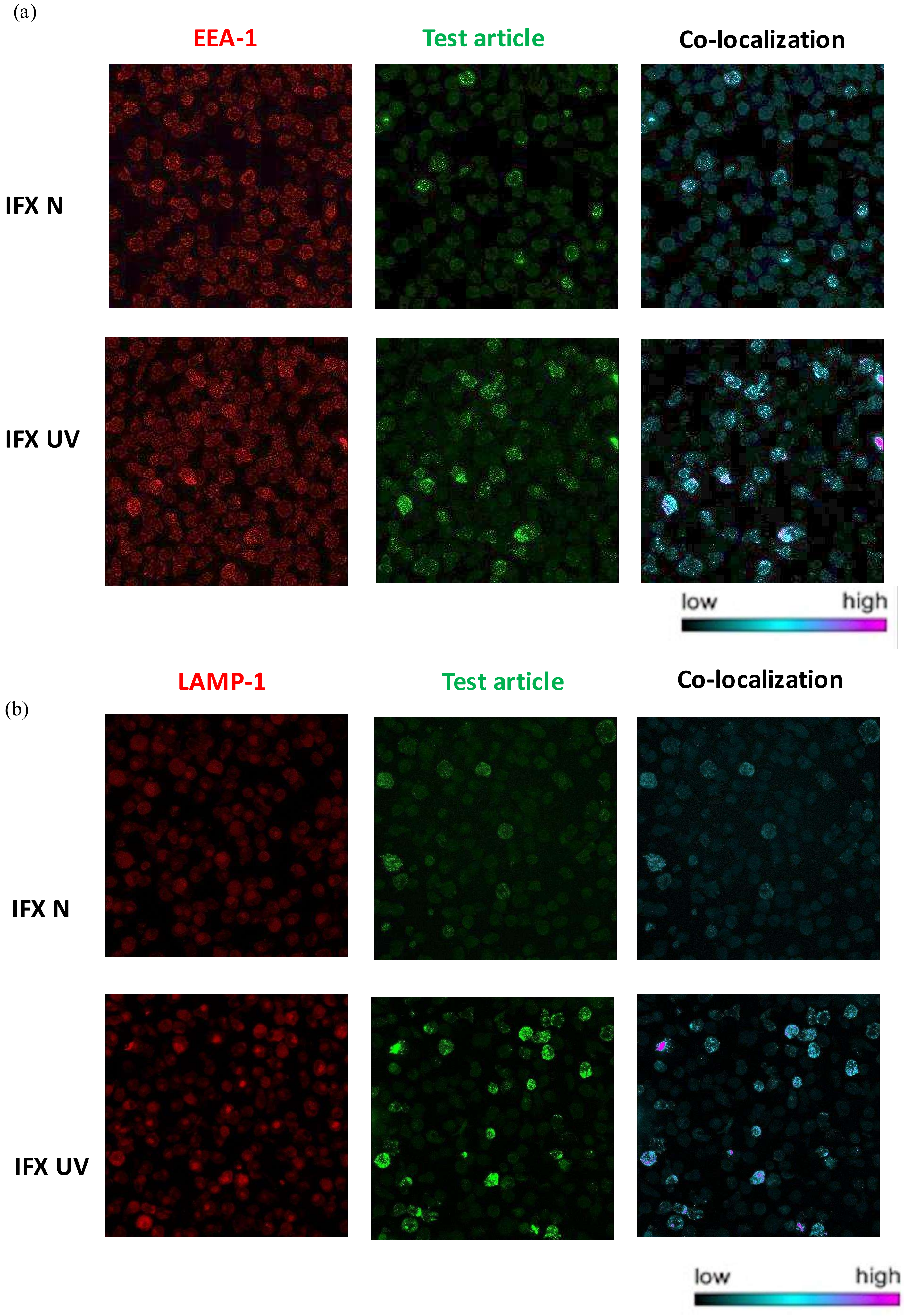
(a)
EEA-1
Test article
Co-localization
IFX N
IFX UV
low
high
(b)
LAMP-1
Test article
Co-localization
IFX N
IFX UV
low
high

(c)

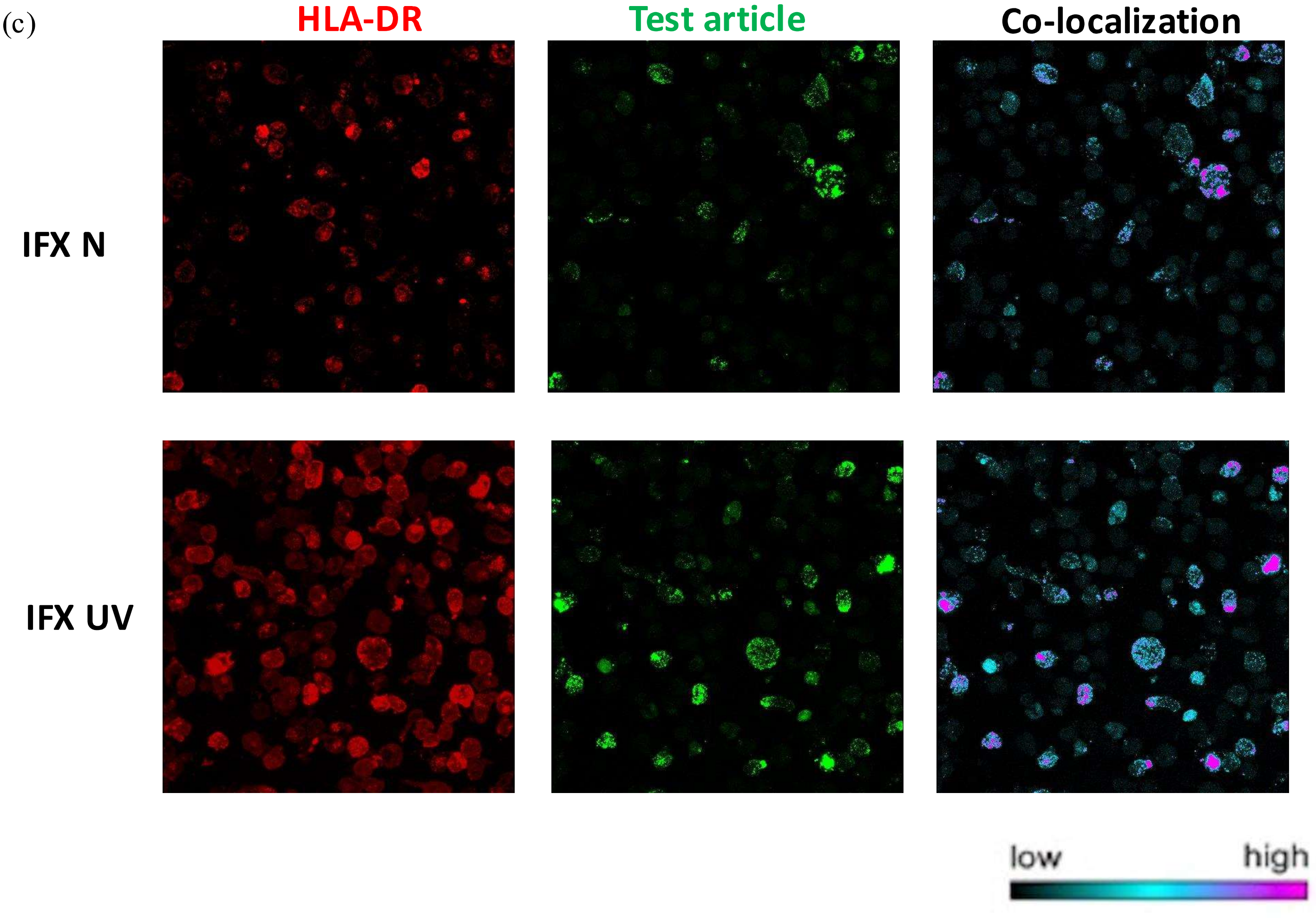

Supplementary data 9: Low magnification images of figure 6b & 7a,d in manuscript.

(a-c) Co-localization of IFX N and IFX UV with EEA-1. moDCs obtained from healthy donors were incubated with IFX N or IFX UV labelled with Alexa-Fluor 488 (green) at 37 °C for 15 minutes (a), 60 minutes (b) or 120 minutes (c). Cells were fixed, permeabilized and immunostained using anti-EEA-1 (red), anti-LAMP-1 (red) or anti-HLA-DR (red) antibodies. Associated fluorescence was detected by laser scanning confocal microscopy at 488-nm (green), 674-nm (red) wavelength.; The color scale bar beneath each image represents the degree of colocalization.
